# Toward a Biologically Plausible Model of LGN-V1 Pathways Based on Efficient Coding

**DOI:** 10.3389/fncir.2019.00013

**Published:** 2019-03-14

**Authors:** Yanbo Lian, David B. Grayden, Tatiana Kameneva, Hamish Meffin, Anthony N. Burkitt

**Affiliations:** ^1^Department of Biomedical Engineering, The University of Melbourne, Melbourne, VIC, Australia; ^2^Centre for Neural Engineering, The University of Melbourne, Melbourne, VIC, Australia; ^3^Faculty of Science, Engineering and Technology, Swinburne University, Melbourne, VIC, Australia; ^4^Department of Optometry and Visual Science, The University of Melbourne, Melbourne, VIC, Australia; ^5^National Vision Research Institute, The Australian College of Optometry, Melbourne, VIC, Australia

**Keywords:** efficient coding, LGN-V1 pathways, biological plausibility, separated ON and OFF sub-regions, push-pull effect, phase-reversed feedback, receptive fields, contrast invariance

## Abstract

Increasing evidence supports the hypothesis that the visual system employs a sparse code to represent visual stimuli, where information is encoded in an efficient way by a small population of cells that respond to sensory input at a given time. This includes simple cells in primary visual cortex (V1), which are defined by their linear spatial integration of visual stimuli. Various models of sparse coding have been proposed to explain physiological phenomena observed in simple cells. However, these models have usually made the simplifying assumption that inputs to simple cells already incorporate linear spatial summation. This overlooks the fact that these inputs are known to have strong non-linearities such the separation of ON and OFF pathways, or separation of excitatory and inhibitory neurons. Consequently these models ignore a range of important experimental phenomena that are related to the emergence of linear spatial summation from non-linear inputs, such as segregation of ON and OFF sub-regions of simple cell receptive fields, the push-pull effect of excitation and inhibition, and phase-reversed cortico-thalamic feedback. Here, we demonstrate that a two-layer model of the visual pathway from the lateral geniculate nucleus to V1 that incorporates these biological constraints on the neural circuits and is based on sparse coding can account for the emergence of these experimental phenomena, diverse shapes of receptive fields and contrast invariance of orientation tuning of simple cells when the model is trained on natural images. The model suggests that sparse coding can be implemented by the V1 simple cells using neural circuits with a simple biologically plausible architecture.

## 1. Introduction

In early experimental studies of cat striate cortex, Hubel and Wiesel found two main types of cells: simple cells and complex cells (Hubel and Wiesel, 1959, 1962). Simple cells exhibit linear spatial summation of visual stimuli, while complex cells have significant non-linear behavior. This difference is reflected in receptive field (RF) structures of the two types of cells. Receptive fields (RFs) describe spatial patterns of light and dark regions in the visual field that in combination are effective at driving neural response. They are frequently modeled as linear spatial filters. Simple cells have a single RF filter, reflecting the linear spatial summation properties, while complex cells pool the output for two or more RF filters in a non-linear fashion.

**Figure 1:**
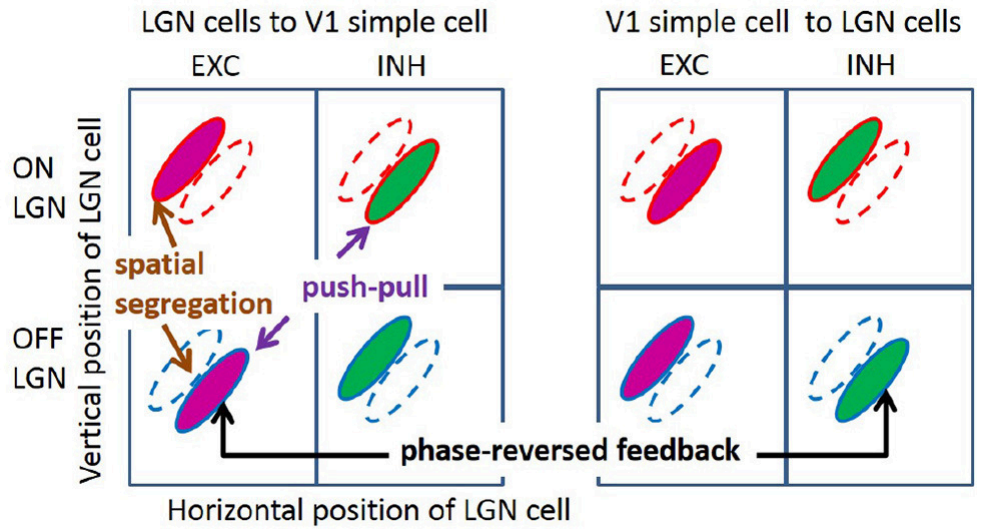
Illustration of segregated ON and OFF sub-regions, the push-pull effect, and phase-reversed feedback. ON and OFF LGN cells are spatially located in a 2D region. The colors of magenta and green represent excitatory and inhibitory connections, respectively.

Over the past decades, some important characteristics of simple cell RF have been observed experimentally (with emphasis on cat and primates, but also ferrets). First, simple cells show a range of selectivity for the orientation of visual stimuli, from highly oriented RFs, which are selective to an optimal orientation, to non-oriented RFs, which are insensitive to orientation. Many RFs of simple cells in V1 are oriented, localized, and bandpass (Hubel and Wiesel, 1962, 1968), while non-orientated RFs are seen in all layers of V1 (Hawken et al., 1988; Chapman and Stryker, 1993). Second, RFs of orientation tuned simple cells can be well-described by two-dimensional Gabor functions (Jones and Palmer, 1987a; Ringach, 2002). In addition, both these studies found some blob-like RFs, which are broadly tuned in orientation. Third, RFs of simple cells have spatially segregated ON and OFF sub-regions (Hubel and Wiesel, 1962; Martinez et al., 2005); i.e., the spatial region that excites the simple cell in response to bright (ON) stimuli is separated from the region that excites the cell in response to dark (OFF) stimuli (left column of Figure 1). Fourth, simple cells show push-pull responses; i.e., if one stimulus excites a simple cell, the stimulus with opposite contrast, but same location, will inhibit the simple cell (Jones and Palmer, 1987b; Ferster, 1988; Hirsch et al., 1998; Martinez et al., 2005). One example of the push-pull effect can be seen on the left of Figure 1 where a simple cell is excited by input from a cell in the lateral geniculate nucleus (LGN) responding to dark spots (an OFF LGN cell) but is effectively inhibited by LGN cells responding a bright spot in the same location (an ON LGN cell). Fifth, feedback from simple cells to LGN cells frequently has a phase-reversed influence compared to the feedforward input (Wang et al., 2006); i.e., where the RF of an ON (OFF) LGN cell is overlapped with the ON (OFF) sub-region of the RF of a simple cell, i.e., feedforward excitation, feedback from the simple cell to the LGN cell is suppressive; where an ON (OFF) LGN cell coincides with the OFF (ON) sub-region of a simple cell RF, i.e., effective feedforward suppression, the feedback is facilitatory. This effect of phase-reversed feedback is also illustrated in Figure 1, where the influence from a simple cell to LGN cells is opposite to the influence from LGN cells to the same simple cell. Lastly, the orientation tuning property of simple cells are contrast invariant; i.e., the shape and width of orientation tuning curves remain the same for different stimulus contrasts (Sclar and Freeman, 1982; Skottun et al., 1987; Finn et al., 2007; Priebe, 2016).

On the other hand, insights from computational modeling of V1 cells have also been used to explain experimental data. Sparse coding has been proposed by many researchers as a principle employed by the brain to process sensory information. Olshausen and Field reproduced localized, oriented and spatially bandpass RFs of simple cells based on a *sparse coding* model that aimed to reconstruct the input with minimal average activity of neurons (Olshausen and Field, 1996, 1997). However, the original model failed to generate non-oriented RFs observed in experiments (Ringach, 2002). Subsequently, Olshausen and colleagues found that the sparse coding model can produce RFs that lack strong orientation selectivity by having many more model neurons than the number of input image pixels (Olshausen et al., 2009). Rehn and Sommer introduced *hard sparseness* to classical sparse coding, which minimizes the number of active neurons rather than the average activity of neurons in the original model, and demonstrated that the modified sparse coding model can generate diverse shapes of simple cell RFs (Rehn and Sommer, 2007). Zhu and Rozell showed that many visual non-classical RF effects of V1 such as end-stopping, contrast invariance of orientation tuning can emerge from a dynamical system based on sparse coding (Zhu and Rozell, 2013).

These studies were important in explaining the RF structure, but made a number of simplifying assumptions that overlooked many details of biological reality, include some or all of the following. First, the responses of neurons (e.g., firing rates) should be non-negative. Second, the learning rule of synaptic connections should be local where the changes of synaptic efficacy depend only on pre-synaptic and post-synaptic responses. Third, the learning rule should not violate Dale's Law, namely that neurons release the same type of transmitter at all their synapses, and consequently, the synapses are either all excitatory or all inhibitory (Strata and Harvey, 1999). Fourth, the computation of the response of any neuron should be local, such that only neurons synaptically connected to this target neuron can be involved. In addition, a biologically plausible model should also be consistent with important experimental evidence. For LGN-V1 visual pathways, experimental evidence includes the existence of a large amount of cortico-thalamic feedback (Swadlow, 1983; Sherman and Guillery, 1996), long-range excitatory but not inhibitory connections between LGN and V1, and separated ON and OFF channels for LGN input (Hubel and Wiesel, 1962; Ferster et al., 1996; Jin et al., 2008, 2011). The original sparse coding model neglects many of the biological constraints described above.

Several recent studies addressed the issue of biological plausibility by incorporating some of these constraints, while continuing to neglect others. For example, Zylberberg and colleagues designed a spiking network (based on sparse coding) that can account for diverse shapes of simple cell RFs using lateral inhibition (Zylberberg et al., 2011). The local learning rule and the use of spiking neurons bring some degree of biological plausibility to the model, but the model employs connections that can change sign during learning, which violates Dale's law, and there are not separate channels for ON and OFF LGN input. Additionally, the effect of sparse coding is achieved by competition between units via lateral inhibition, but a recent study suggested that dominant lateral interactions are excitatory in the mouse cortex (Lee et al., 2016). In another modeling work of simple cell RFs, Wiltschut and Hamker designed an efficient coding model with separated ON and OFF LGN cells, and, feedforward, feedback, and lateral connections that can generate various types of simple cell RFs (Wiltschut and Hamker, 2009), but their model does not incorporate Dale's law.

As with earlier studies (Olshausen and Field, 1996, 1997; Rehn and Sommer, 2007; Olshausen et al., 2009), these more recent studies (Wiltschut and Hamker, 2009; Zylberberg et al., 2011), incorporating biological constraints, have continued to focus on the RF structure of simple cells, while largely neglecting the experimental phenomena shown in Figure 1. This is because they have typically not separated inputs from ON and OFF LGN cells, which is a key distinction underlying all the phenomena listed in Figure 1. One important question in this regard is how these non-linear (half-wave rectified) LGN inputs are combined to give linear RFs for simple cells and whether this causes the experimental phenomena listed in Figure 1. To our knowledge, Jehee and Ballard are the only researchers that have explicitly explained the effect of phase-reversed feedback using a model based on predictive coding (Jehee and Ballard, 2009). However, the RFs generated by their model do not match well with those observed in experiments and the push-pull effect for simple cells has not been explained. In addition, the formula for calculating responses of model neurons (Jehee and Ballard, 2009, Equation 7) is not local and the learning rule neglects Dale's law.

In this paper, we propose a two-layer model of LGN-V1 visual pathways that can account for experimental phenomena:

- Segregated ON and OFF sub-regions of simple cells,
- The push-pull effect for simple cells,
- Phase-reversed cortico-thalamic feedback,
- Diverse shapes of RFs (oriented and non-oriented),
- Contrast invariance of orientation tuning.

Our model is biologically plausible by incorporating:

- Separate channels of ON and OFF LGN input,
- Non-negative neural responses,
- Local learning rule,
- Dale's law,
- Local computation,
- Dynamics of rate-based model neurons,
- Feedback from V1 to LGN.

The first layer consists of ON and OFF LGN cells and the second layer consists of simple cells. The connections from the first layer to the second layer (feedforward connections) and from the second layer to the first layer (feedback connections) consist of separate excitatory and inhibitory connections. Even though the inhibitory connections between LGN and V1 should be implemented via intermediate populations of inhibitory interneurons, we use neurons that have both excitatory and inhibitory connections to simplify the circuit. This aspect of the model is not biologically plausible, but possible biologically plausible neural circuits for implementing inhibitory connections are proposed in the Discussion section. The model presented here is relevant to visual cortices both with and without an orientation columnar organization.

The novelty of the model proposed here is that it models LGN-V1 pathways using segregated ON and OFF LGN channels and separate excitatory and inhibitory connections to investigate the structure of connections between LGN and simple cells to explain a wide range of experimental phenomena. In addition, it can generate a wide variety of experimentally observed RFs of simple cells. Also, the model is biologically plausible by respecting many biological constraints and important experimental evidence. Finally, the experimental phenomena explained in this paper are all caused by the structure of learned connections between LGN and V1 after the model is trained on natural image data.

## 2. Materials and Methods

### 2.1. Sparse Coding

The original sparse coding model (Olshausen and Field, 1996) proposed that simple cells represent their sensory input in such a way that their spiking rates in response to natural images tend to be statistically independent and rarely attain large values (near the top of the cells' dynamic range). Mathematically this means that the joint distribution of spike rates over natural images is the product of the distributions for individual cells, and that each of these individual distributions has a long tail (i.e., high kurtosis). Additionally it was proposed that the representation should allow the reconstruction of the sensory input through a simple weighted sum of visual features with minimal error. This can be formulated as an optimization problem of minimizing the cost function,

(1) $$\begin{array}{l} E(A,s)=\frac{1}{2}\|x-As{\|}_{2}^{2}+\lambda \sum_{i}Q(s_{i}), \end{array}$$

where **x** represents the input, columns of the matrix **A** represent basis vectors that are universal visual features from which any image can be constructed from a weighted sum, **s** is the vector of responses, *s*_*i*_, of model units that represent the corresponding coefficients for all basis vectors, *Q*(·) represents a penalty function that favors low activity of model units, and λ is a parameter that scales the penalty function (Olshausen and Field, 1996, 1997). The term **As** in Equation (1) is the reconstruction of the input from the model, so the first term on the right-hand-side of Equation (1) represents the sum of squared difference between the input and model reconstruction. The second term on the right-hand-side of Equation (1) tends to push **s** to small values. Therefore, by solving this minimization problem, the model finds a sparse representation for the input. By taking the partial derivatives of Equation (1) in terms of the elements of **A** and **s**, and applying gradient descent, the dynamic equations and the learning rule are given by

(2) $$\begin{array}{l} \;\dot{s}=A^{T}r-\lambda Q\prime (s)\\ \Delta A\propto \langle rs^{T}\rangle , \end{array}$$

where **r** = **x** − **As**, 〈·〉 is the average operation, the dot notation represents differentiation with regard to time, and *Q*′(·) represents the derivative of *Q*(·).

**Figure 2:**
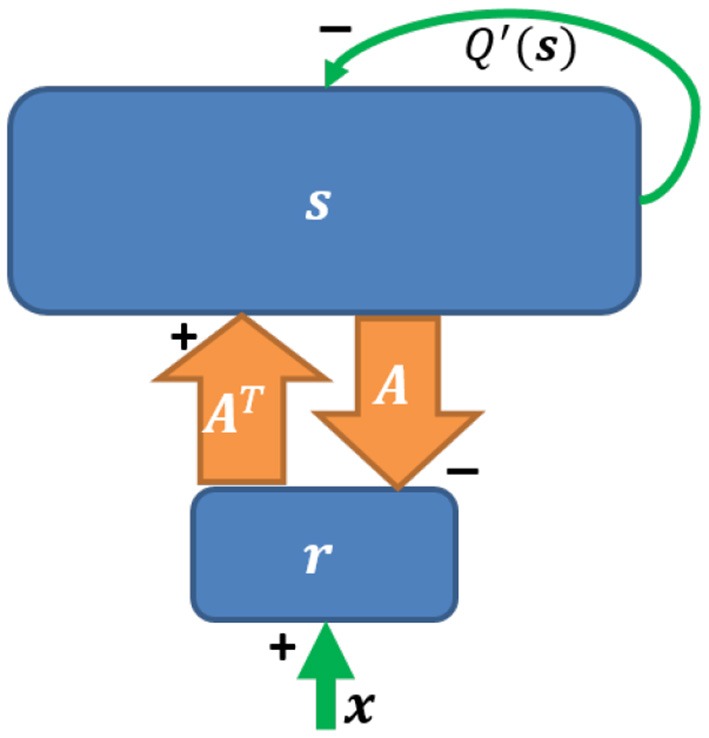
The network implementation of sparse coding. Upward and downward arrows represent feedforward and feedback connections. The reconstruction **As** is subtracted via negative feedback. *Q*′(**s**) represents self-inhibition of neurons (Adapted from **Figure 5** in Olshausen and Field, 1997).

Based on Equation (2), a network implementation of sparse coding, shown in Figure 2, was proposed by Olshausen and Field (1997) who suggested that a feedforward-feedback loop can implement sparse coding. The input to the model was natural images that had been whitened using a filter that resembles the center-surround structure of retinal ganglion RFs. However, the original sparse coding model was not biologically plausible in several aspects, such as the possibility of negative spiking rates and the violation of Dale's law. In addition, the input the the model was not split into separate ON and OFF channels. Finally, this network imposed feedback synaptic connections that were anti-symmetric to the corresponding feedforward connections (i.e., equal but opposite in sign) and it was unclear how such symmetry could be achieved using biologically plausible mechanisms.

### 2.2. Structure of Our Model

**Figure 3:**
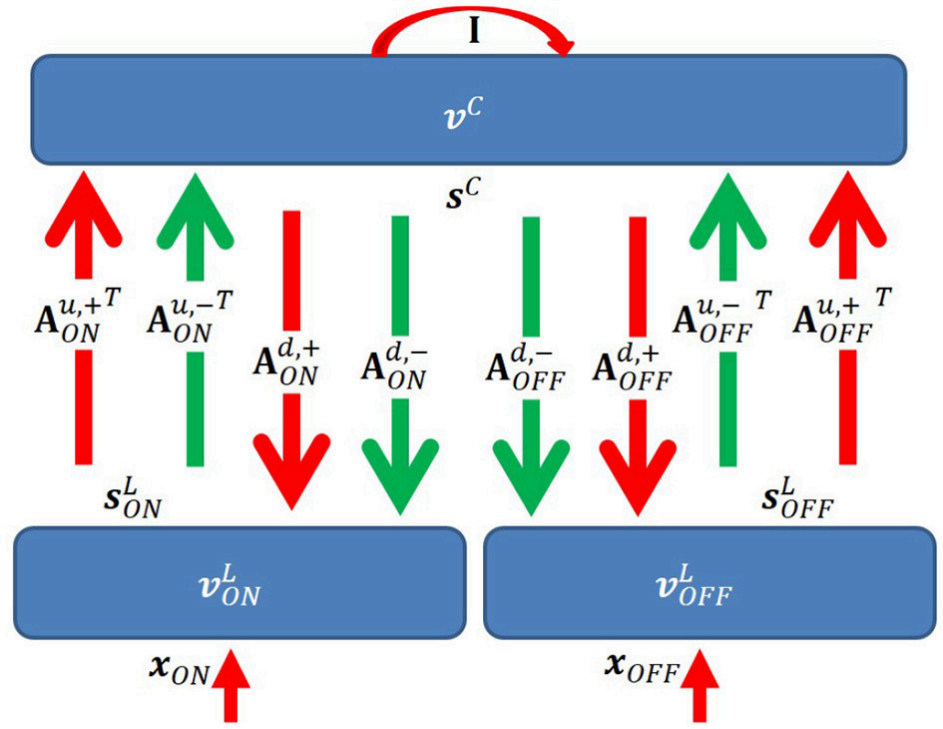
Graphical representation of the model. **I** is the identity matrix that represents self-excitation. Red and green arrows represent excitatory and inhibitory connections, respectively. Upward and downward arrows are for feedforward and feedback pathways. Notation defined in the main text.

We propose a two-layer network with rate-based neurons that models the activities of LGN cells (first layer), and simple cells (second layer), respectively (Figure 3). The model is based on a locally competitive algorithm that efficiently implements sparse coding with neural dynamics with non-negative spiking rates (Rozell et al., 2008).

We first define the parameters of the model that will be used throughout the paper. A summary of all symbols defined below is shown in Table 1. There are 2*N* LGN cells in the first layer, with *N* ON LGN cells and *N* OFF LGN cells, and *M* simple cells in the second layer. Denote $\text{x}={\left[x_{1},\cdots \;,x_{2N}\right]}^{T}$ as the vector of input stimuli to the first layer. Denote **x**_ON_ as the input to ON LGN cells (the first *N* elements of **x**) and **x**_OFF_ as the input to OFF LGN cells (the last *N* elements of **x**), i.e., $\text{x}={\left[{\text{x}}_{\text{ON}}^{T},{\text{x}}_{\text{OFF}}^{T}\right]}^{T}$.

**Table 1:** Model symbols.

**Description**	**Symbol**
Input stimuli to LGN cells	**x**
Input stimuli to ON LGN cells	**x**_ON_
Input stimuli to OFF LGN cells	**x**_OFF_
Membrane time constant of LGN cells (12 ms)	τ_L_
Membrane potentials of LGN cells	**v**^L^
Membrane potentials of ON LGN cells	${\text{v}}_{\text{ON}}^{\text{L}}$
Membrane potentials of OFF LGN cells	${\text{v}}_{\text{OFF}}^{\text{L}}$
Firing rates of LGN cells	**s**^L^
Firing rates of ON LGN cells	${\text{s}}_{\text{ON}}^{\text{L}}$
Firing rates of OFF LGN cells	${\text{s}}_{\text{OFF}}^{\text{L}}$
Spontaneous firing rate of LGN cells (2 Hz)	*s*_b_
Membrane time constant of cortical simple cells (12 ms)	τ_C_
Membrane potentials of cortical simple cells	**v**^C^
Leakage voltages of cortical simple cells	${\text{v}}_{\text{leak}}^{\text{C}}$
Firing rates of cortical simple cells	**s**^C^
Excitatory connection: all LGN cells to simple cells	**A**^u,+^
Excitatory connection: ON LGN cells to simple cells	${\text{A}}_{\text{ON}}^{\text{u},+}$
Excitatory connection: OFF LGN cells to simple cells	${\text{A}}_{\text{OFF}}^{\text{u},+}$
Inhibitory connection: all LGN cells to simple cells	**A**^u,−^
Inhibitory connection: ON LGN cells to simple cells	${\text{A}}_{\text{ON}}^{\text{u},-}$
Inhibitory connection: OFF LGN cells to simple cells	${\text{A}}_{\text{OFF}}^{\text{u},-}$
Excitatory connection: simple cells to all LGN cells	**A**^d,+^
Excitatory connection: simple cells to ON LGN cells	${\text{A}}_{\text{ON}}^{\text{d},+}$
Excitatory connection: simple cells to OFF LGN cells	${\text{A}}_{\text{OFF}}^{\text{d},+}$
Inhibitory connection: simple cells to all LGN cells	**A**^d,−^
Inhibitory connection: simple cells to ON LGN cells	${\text{A}}_{\text{ON}}^{\text{d},-}$
Inhibitory connection: simple cells to OFF LGN cells	${\text{A}}_{\text{OFF}}^{\text{d},-}$
Sparsity level (0.6)	λ
Learning rate	η

Denote **v**^L^ and **s**^L^ as 2*N* × 1 vectors that represent membrane potentials and firing rates of LGN cells in the first layer. Denote ${\text{v}}_{\text{ON}}^{\text{L}}$, ${\text{s}}_{\text{ON}}^{\text{L}}$, ${\text{v}}_{\text{OFF}}^{\text{L}}$, and ${\text{s}}_{\text{OFF}}^{\text{L}}$ as *N* × 1 vectors that represent the membrane potentials and firing rates of ON and OFF LGN cells, i.e., ${\text{v}}^{\text{L}}={\left[{{\text{v}}_{\text{ON}}^{\text{L}}}^{T},{{\text{v}}_{\text{OFF}}^{\text{L}}}^{T}\right]}^{T}$ and ${\text{s}}^{\text{L}}={\left[{{\text{s}}_{\text{ON}}^{\text{L}}}^{T},{{\text{s}}_{\text{OFF}}^{\text{L}}}^{T}\right]}^{T}$. Similarly, **v**^C^ and **s**^C^ are *M* × 1 vectors that represent membrane potentials and firing rates of *M* cortical simple cells in the second layer.

In our model, there are several important connections: feedforward (up) excitatory and inhibitory connections from LGN cells to simple cells, feedback (down) excitatory and inhibitory connections from simple cells to LGN cells, and self-excitatory connections of simple cells that represent self-excitation. Definitions of connections are described below. One aspect of the model that lacks biological plausibility is existence of inhibitory connections between thalamus and cortex, but we propose biologically plausible neural circuits of implementing this aspect of the model in the Discussion section.

Denote ${\text{A}}_{\text{ON}}^{\text{u},+}$ as an *N* × *M* matrix with non-negative elements that represents the feedforward excitatory connections from ON LGN cells to simple cells. Each column of ${\text{A}}_{\text{ON}}^{\text{u},+}$ represents connections from *N* ON LGN cells to a simple cell. Similarly, denote ${\text{A}}_{\text{OFF}}^{\text{u},+}$ as an *N* × *M* matrix with non-negative elements that represents the feedforward excitatory connections from OFF LGN cells to simple cells. Denote ${\text{A}}_{\text{ON}}^{\text{u},-}$ and ${\text{A}}_{\text{OFF}}^{\text{u},-}$ as *N* × *M* matrices with non-positive elements that represent inhibitory connections from ON and OFF LGN cells to simple cells, respectively. Denote **A**^u,+^ and **A**^u,−^ as 2*N* × *M* matrices that represents all excitatory and inhibitory connections from LGN to V1; then we have ${\text{A}}^{\text{u},+}=\left[{\text{A}}_{\text{ON}}^{\text{u},+}\;{\text{A}}_{\text{OFF}}^{\text{u},+}\right]$ and ${\text{A}}^{\text{u},-}=\left[{\text{A}}_{\text{ON}}^{\text{u},-}\;{\text{A}}_{\text{OFF}}^{\text{u},-}\right]$.

For the feedback pathway, similar notation is used except superscript “d” represents feedback connections from simple cells to LGN cells. Therefore, we have ${\text{A}}^{\text{d},+}=\left[{\text{A}}_{\text{ON}}^{\text{d},+}\;{\text{A}}_{\text{OFF}}^{\text{d},+}\right]$ and ${\text{A}}^{\text{d},-}=\left[{\text{A}}_{\text{ON}}^{\text{d},-}\;{\text{A}}_{\text{OFF}}^{\text{d},-}\right]$.

Using the notation defined above, the dynamics of ON and OFF LGN cells located in the first layer are given by

(3) $$\begin{array}{l} \tau_{\text{L}}{\dot{v}}_{\text{ON}}^{\text{L}}\;=-v_{\text{ON}}^{\text{L}}+x_{\text{ON}}+A_{\text{ON}}^{\text{d},\text{+}}s^{\text{C}}+A_{\text{ON}}^{\text{d},\text{−}}s^{\text{C}}+s_{\text{b}}\\ \;s_{\text{ON}}^{\text{L}}=\max(v_{\text{ON}}^{\text{L}},0) \end{array}$$

and

(4) $$\begin{array}{l} \tau_{\text{L}}{\dot{v}}_{\text{OFF}}^{\text{L}}=-v_{\text{OFF}}^{\text{L}}+x_{\text{OFF}}+A_{\text{OFF}}^{\text{d},\text{+}}s^{\text{C}}+A_{\text{OFF}}^{\text{d},-}s^{\text{C}}+s_{\text{b}},\\ \;s_{\text{OFF}}^{\text{L}}=\max(v_{\text{OFF}}^{\text{L}},0), \end{array}$$

where τ_L_ is the time constant of the membrane potentials of LGN cells, *s*_b_ is a constant that represents the instantaneous firing rate of the background input (i.e., from neurons outside the network), and the max operation represents the firing dynamics such that a cell only fires when the membrane potential is above a threshold.

Therefore, using the combined notation for ON and OFF LGN cells, the dynamics of LGN cells can be written as

(5) $$\begin{array}{l} \tau_{\text{L}}{\dot{v}}^{\text{L}}=-v^{\text{L}}+x+(A^{\text{d},\text{+}}+A^{\text{d},-})s^{\text{C}}+s_{\text{b}}\\ \;s^{\text{L}}=\max(v^{\text{L}},0). \end{array}$$

The dynamics of simple cells located in the second layer is given by

(6) $$\begin{array}{l} \tau_{\text{C}}{\dot{v}}^{\text{C}}=-(v^{\text{C}}-v_{\text{leak}}^{\text{C}})+A_{\text{ON}}^{u,+T}s_{\text{ON}}^{\text{L}}+A_{\text{ON}}^{u,-T}s_{\text{ON}}^{\text{L}}\\ \;+A_{\text{OFF}}^{u,+T}s_{\text{OFF}}^{\text{L}}+A_{\text{OFF}}^{u,-T}s_{\text{OFF}}^{\text{L}}+s^{\text{C}}, \end{array}$$

which can be reformulated as

(7) $$\begin{array}{l} \tau_{\text{C}}{\dot{v}}^{\text{C}}=-v^{\text{C}}+v_{\text{leak}}^{\text{C}}+{\left(A^{\text{u},\text{+}}+A^{\text{u},-}\right)}^{T}s^{\text{L}}+s^{\text{C}}\\ \;s^{\text{C}}=\max(v^{\text{C}}-\lambda ,0), \end{array},$$

where τ_C_ is the time constant of the membranes of simple cells and λ is the threshold of the rectifying function of firing rates. In addition, λ is a positive constant that introduces sparseness into the model, **s**^C^ represents the self-excitation of simple cells, which comes from reformulating the model equations of the locally competitive algorithm (Rozell et al., 2008), and ${\text{v}}_{\text{leak}}^{\text{C}}$ represents the change of membrane potential caused by leakage currents. The leakage currents drive the membrane potentials of simple cells to their resting potentials when there is no external input, i.e., **v**^C^ is zero. Therefore, the steady states of the model dynamics are ${\text{v}}^{\text{L}}=s_{\text{b}}$, ${\text{s}}^{\text{L}}=s_{\text{b}}$, **v**^C^ = 0, and **s**^C^ = 0, which implies that ${\text{v}}_{\text{leak}}^{\text{C}}=-{\left({\text{A}}^{\text{u},+}+{\text{A}}^{\text{u},-}\right)}^{T}{\text{s}}_{\text{b}}$, where **s**_b_ is a vector whose elements are all equal to *s*_b_. Equations 5 and 7 are solved simultaneously by iteration to obtain values of membrane potentials and firing rates.

The codes to run the model are available from ModelDB (http://modeldb.yale.edu/247970).

### 2.3. Learning Rule

The learning process of the model is based on a Hebbian or anti-Hebbian rule, namely that the change of synaptic strength is related only to local pre-synaptic and post-synaptic activities.

The learning rules are given by

(8) $$\begin{array}{l} \Delta A^{\text{u},\text{+}}=\eta \langle (s^{\text{L}}-s_{\text{b}})s^{{\text{C}}^{T}}\rangle \\ \Delta A^{\text{u},-}=\eta \langle (s^{\text{L}}-s_{\text{b}})s^{{\text{C}}^{T}}\rangle \\ \Delta A^{\text{d},\text{+}}=-\eta \langle (s^{\text{L}}-s_{\text{b}})s^{{\text{C}}^{T}}\rangle \\ \Delta A^{\text{d},-}=-\eta \langle (s^{\text{L}}-s_{\text{b}})s^{{\text{C}}^{T}}\rangle , \end{array}$$

where η is the learning rate, 〈·〉 is the ensemble average operation over some samples, ${\text{s}}^{\text{L}}-s_{\text{b}}$ is the vector such that each element of vector **s**^L^ is subtracted by scalar *s*_b_, and $\left({\text{s}}^{\text{L}}-s_{\text{b}}\right){{\text{s}}^{\text{C}}}^{T}$ is the matrix given by the outer product of vectors ${\text{s}}^{\text{L}}-s_{\text{b}}$ and **s**^C^.

The change of synaptic strength depends only on the pre-synaptic activity (**s**^L^) and post-synaptic activity (**s**^C^). Therefore, this learning rule is local and thus biophysically realistic. In obedience to Dale's law, all the weights of **A**^u,+^ and **A**^d,+^ are kept non-negative and all weights of **A**^u,−^ and **A**^d,−^ are kept non-positive during learning. If any synaptic weight changes sign after applying Equation (8), the synaptic weight is set to zero. In addition, after each learning iteration, synaptic weights are multiplicatively normalized to ensure that Hebbian learning is stable. Specifically, each column of **A**^u,+^ and **A**^d,−^ is normalized to norm *l*_1_ and each column of **A**^u,−^ and **A**^d,+^ is normalized to norm *l*_2_. The multiplicative normalization of synaptic weights may be achieved by homeostatic mechanisms (Turrigiano, 2011), but these are not implemented here as they are not the focus of this paper.

### 2.4. Input

The data set used in our simulation consists of 10 pre-whitened 512 × 512 pixel images of natural scenes provided by Olshausen and Field Olshausen and Field (1996). Some previous studies of sparse coding (efficient coding) also used this data set (Olshausen and Field, 1996; Wiltschut and Hamker, 2009; Zylberberg et al., 2011; Zhu and Rozell, 2013). The input stimuli to the model are chosen to be 16 × 16 pixel image patches sampled from these 10 pre-whitened 512 × 512 pixel images, similar to previous studies (Zylberberg et al., 2011; Zhu and Rozell, 2013).

**Figure 4:**
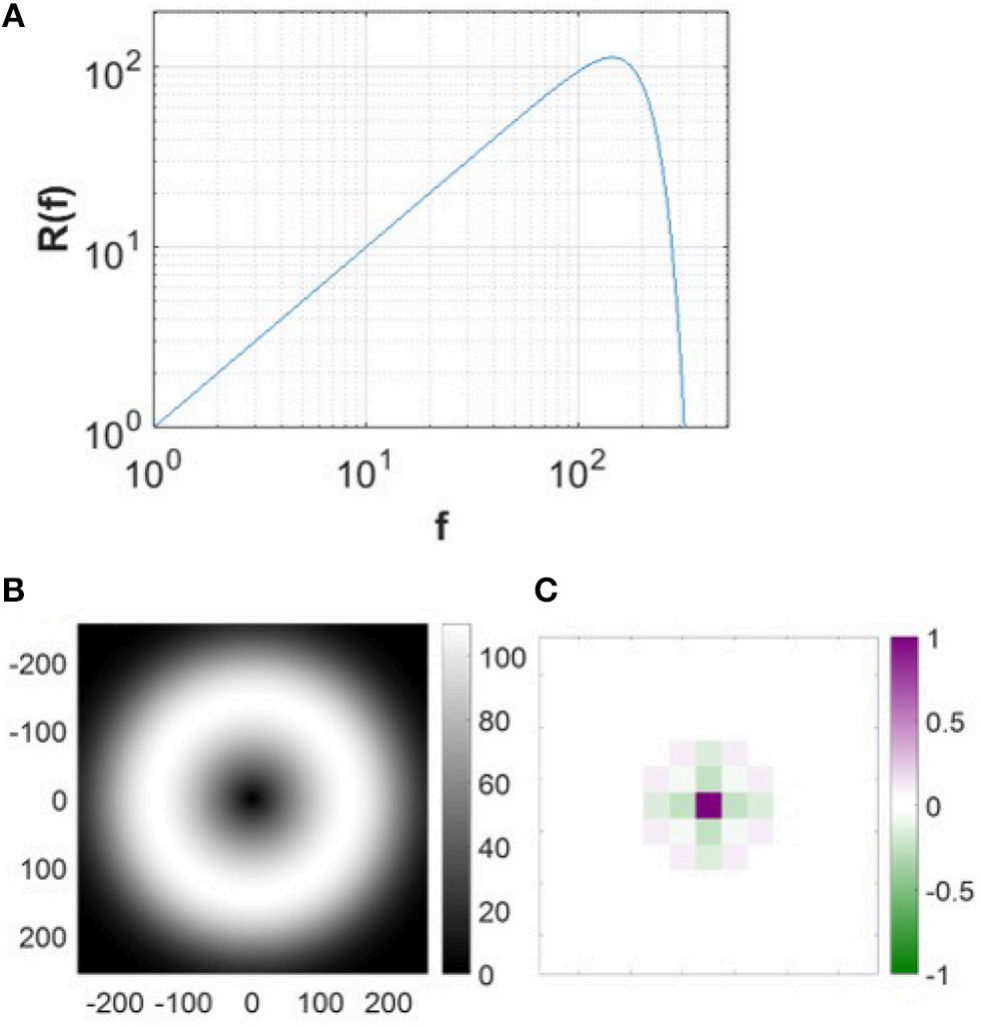
Pre-whitening filter. **(A)** The pre-whitening filter described in Equation (9). **(B)** The pre-whitening filter in 2D frequency domain. **(C)** The spatial profile of the pre-whitening filter. The scale of the spatial filter is arbitrarily normalized to convert the luminance to the membrane potential relative to the maximal luminance of the image.

The pre-whitening process mimics the spatial filtering of retinal processing up to a cut-off frequency determined by the limits of visual acuity (Atick and Redlich, 1992). This process is realized by passing the original natural images through a zero-phase whitening filter with root-mean-square power spectrum,

(9) $$\begin{array}{l} R(f)=fe^{-{\left(f/f_{c}\right)}^{4}}, \end{array}$$

where *f*_*c*_ = 200 cycles/picture (Olshausen and Field, 1997). Figure 4 shows the spatial and frequency profiles of the pre-whitening filter. The spatial profile of the filter (Figure 4C), obtained by taking the 2D inverse Fourier transform of the filter in the 2D frequency domain, approximates center-surround RFs of LGN cells in a pixel image. The pre-whitening filter described in Equation (9) is widely used in computational studies (Jehee et al., 2006; Jehee and Ballard, 2009; Wiltschut and Hamker, 2009; Zhu and Rozell, 2013).

The pre-whitened images are then scaled to variance 0.2 similar to Olshausen and Field (1997). Image patches are fed into the first layer, which consists of *N* ON LGN cells and *N* OFF LGN cells, i.e., one pixel is fed into one ON LGN cell and one OFF LGN cell. If a pixel intensity in a pre-whitened image patch is negative, we assign the absolute value of the pixel intensity to the input of the OFF LGN cell and set the input of the corresponding ON LGN cell to zero; if the pixel intensity is positive, we set the input of the ON LGN cell to the pixel intensity and set the input to the OFF LGN cell to zero.

### 2.5. Training

Since we use 16 × 16 pixel images as the input to our model, 256 ON and 256 OFF LGN cells (*N* = 256) are required in the first layer. We use 256 simple cells (*M* = 256) in the second layer. The first-order Euler method is implemented to solve the dynamical system described by Equation 5 and 7. We choose a time scale in which the passive membrane time constant is τ_L_ = τ_C_ = 12 ms, within the physiological range (Dayan et al., 2001), and sparsity level λ = 0.6 similar to Zhu and Rozell (2013). The spontaneous firing rate, *s*_b_, is chosen as *s*_b_ = 2 Hz, the median of spontaneous firing rates of the mouse LGN cells in the experimental study of Tang et al. (2016). There are 30 integration time steps, with an integration time step of 3ms, for calculating neuronal responses per stimulus with the assumption that neural responses will converge after 30 iterations.

Learning rules in Equation (8) are used to update the synaptic weights. For the normalization step after each learning iteration, each column of **A**^u,+^ and **A**^d,−^ is normalized to have norm *l*_1_ and each column of **A**^u,−^ and **A**^d,+^ is normalized to have norm *l*_2_. Elements of **A**^u,+^ and **A**^d,+^ are non-negative and initialized randomly using an exponential distribution with mean 0.5. **A**^u,−^ and **A**^d,−^ are initialized randomly with non-positive elements that are sampled from an exponential distribution with mean −0.5. Then, synaptic weights are normalized before the learning process starts. Results shown in this paper are from simulations with *l*_1_ = *l*_2_ = 1 (unit norm), as used in the previous study by Rozell et al. (2008). The learning rule based on the average activities of a mini-batch is applied; i.e., in every epoch, a mini-batch that consists of 100 randomly selected 16 × 16 pixel images sampled from the data set is used. Before the training process of natural image patches, the model is pre-trained on white noise for 10, 000 epochs to mimic the process of pre-development of the visual system; the learning rate is 0.5 in pre-training. To ensure that the weights converge after learning on natural image patches, we use 30, 000 epochs in the training process, where the learning rate is 0.5 for the first 10, 000 epochs, 0.2 for the second 10, 000 epochs and 0.1 for the third 10, 000 epochs. Learning rates were chosen to ensure stable convergence of the weights in a reasonable time; but the results are not sensitive to moderate changes.

### 2.6. Recovering Receptive Fields of Model Simple Cells Using White Noise

In order to estimate the RFs of model simple cells in a systematic way, we use the method of spike-triggered averaging to find the pattern that each simple cell responds to on average (Schwartz et al., 2006). Using *K* 16 × 16 white noise stimuli **n**_1_, ···, **n**_*K*_, we present pre-processed stimuli to the model, record the firing rates of a simple cell, *s*_1_, ···, *s*_*K*_, and then estimate the RF, **F**, of the simple cell as the weighted average,

(10) $$\begin{array}{l} F=\frac{s_{1}n_{1}+\cdots +s_{K}n_{K}}{s_{1}+\cdots +s_{K}}. \end{array}$$

We used 70, 000 white noise stimuli, i.e., *K* = 70, 000.

In our simulations, we have two versions of estimated RFs using the two different methods of pre-processing the white noise stimuli: the same pre-whitening filter for natural scenes (Equation 9) and a low-pass filter described by

(11) $$\begin{array}{l} L(f)=e^{-{\left(f/f_{s}\right)}^{4}}. \end{array}$$

### 2.7. Fitting Receptive Fields to Gabor Functions

The RFs of visual cortical cells are often modeled using a 2D Gabor function *G*(*x, y*) of the form

(12) $$\begin{array}{l} G(x,y;x_{0},y_{0},\sigma_{x},\sigma_{y},f_{s},\beta ,\theta ,\phi )\\ \;=\beta \cos(2\pi f_{s}x^{\prime }+\phi )e^{-{\left(\frac{x^{\prime }}{\sqrt{2}\sigma_{x}}\right)}^{2}-{\left(\frac{y^{\prime }}{\sqrt{2}\sigma_{y}}\right)}^{2}} \end{array}$$

with

(13) $$\begin{array}{l} x^{\prime }=(x-x_{0})\cos\theta +(y-y_{0})\sin\theta \\ y^{\prime }=-(x-x_{0})\sin\theta +(y-y_{0})\cos\theta , \end{array}$$

where β is the amplitude, (*x*_0_, *y*_0_) is the center, σ_*x*_ and σ_*y*_ are standard deviations of the Gaussian envelope, θ is the orientation, *f*_*s*_ is the spatial frequency, and ϕ is the phase of the sinusoid wave (Ringach, 2002). These parameters are fitted using the built-in MATLAB (version R2016b, MathWorks, MA, USA) function, *lsqcurvefit*, that efficiently solves non-linear curve-fitting problems using a least-squares method. The fitting error is defined as the square of the ratio between the fitting residual and RF.

To ensure that results were only reported for RFs that were well-fitted to Gabor functions, we excluded RFs for which either (1) the synaptic fields had fitting error larger than 40% or (2) the center of the fitted Gabor functions lay either outside the block, or within one standard deviation of the Gaussian envelope of the block edge (Zylberberg et al., 2011). After applying these two quality control measures, 140 out of 256 model cells remained for subsequent analysis.

### 2.8. Measuring the Overlap Index Between ON and OFF Sub-regions

To investigate the extent of overlap between ON and OFF sub-regions, we used an overlap index that was used in experimental studies (Schiller et al., 1976; Martinez et al., 2005). Similar to the method used in Martinez et al. (2005), each ON and OFF excitatory sub-region was fitted by an elliptical Gaussian function:

(14) $$\begin{array}{l} h(x,y;x_{0},y_{0},a,b,\theta ,\gamma )=\frac{\gamma }{2\pi ab}e^{-\frac{{x^{\prime }}^{2}}{2a^{2}}-\frac{{y^{\prime }}^{2}}{2b^{2}}} \end{array}$$

where γ is the amplitude, *a* and *b* are half axes of the ellipse, and *x*′ and *y*′ are the transformed coordinates given by Equation (13). If there are more than one ON (or OFF) sub-regions for the simple cell, only the most significant sub-region was fitted by the elliptic Gaussian. If either the ON or OFF sub-region of a simple cell has fitting error larger than 40% or has the half axis, *a*, larger than 3 pixels, this simple cell is excluded. 92 simple cells remained for the analysis of overlap index.

The overlap index, *I*_o_, is then defined as

(15) $$\begin{array}{l} I_{\text{o}}=\frac{W_{\text{ON}}+W_{\text{OFF}}-d}{W_{\text{ON}}+W_{\text{OFF}}+d},\;(-1<I_{\text{o}}\leq 1) \end{array}$$

where *W*_ON_ and *W*_OFF_ are the half width measured at the point where the response is 30% of the maximal response, and *d* is the distance between the centers of ON and OFF sub-regions. Smaller values of *I*_o_ indicate more segregation between ON and OFF sub-regions.

### 2.9. Measuring the Push-Pull Index

The push-pull effect of the model was measured by a push-pull index (Martinez et al., 2005). First, for each simple cell, we recorded the membrane potential, *P*, when the preferred input (the synaptic field) was presented to the model. Next, we recorded the membrane potential, *N*, while presenting the opposite of preferred input to the model. To make the measurement independent of the relative strength of different simple cells, *P* and *N* were normalized by

(16) $$\begin{array}{l} P=\frac{P}{\max(|P|,|N|)}\text{ and }N=\frac{N}{\max(|P|,|N|)}. \end{array}$$

The Push-pull index, *I*_p_, is then defined as

(17) $$\begin{array}{l} I_{\text{p}}=|P+N|,\;(0\leq I_{\text{p}}\leq 2). \end{array}$$

Smaller values of *I*_p_ indicate stronger push-pull effect.

### 2.10. Measuring Contrast Invariance of Orientation Tuning

The method in (Zhu and Rozell, 2013) was used to investigate contrast invariance of orientation tuning and the procedure is as follows. First, an exhaustive search was performed to find the preferred circular sinusoidal grating in the parameter space of the following ranges: radius of the grating was between 1 pixel and 2.5min(σ_*x*_, σ_*y*_) (smaller than 8 pixels which is the maximum radius for a 16 × 16 image patch) with the stepsize of 1 pixel; spatial frequency was between 0.05 and 0.3 cycles/pixel with the stepsize of 0.05 cycles/pixel; orientation was between 0 and 180 degrees with the stepsize of 5 degrees; phase was between 0 and 360 degrees with the stepsize of 30 degrees. Next, we measured the mean response to the drifting grating with orientations between 0 and 180 degrees with the stepsize of 5 degrees while varying the contrast of the stimuli from 0.2 to 1 in increments of 0.2, where contrast is defined as the amplitude of the sinusoidal grating. The orientation tuning curve for each contrast level was then fit to the Gaussian function and the half-height bandwidth of the Gaussian fit was calculated. The slope of the linear fit to half-height bandwidth vs. contrast for the cell was used to plot the population statistics of contrast invariance (Alitto and Usrey, 2004). Here, only 68 model simple cells that have oriented RFs located well within the 16 × 16 image patch were selected for the analysis.

## 3. Results

**Figure 5:**
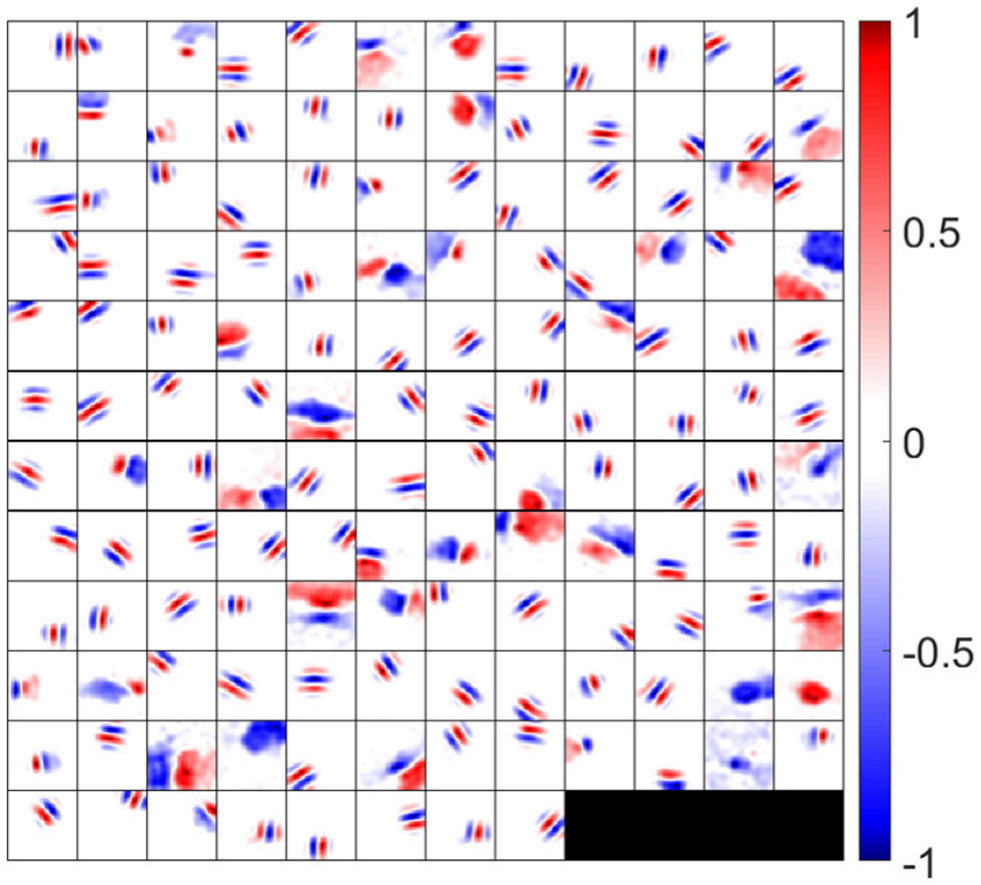
Synaptic fields (defined in Equation 18) for 140 selected simple cells. Each block is a 16 × 16 image that represents the combined effects of ON and OFF LGN cells for a simple cell in spatial domain. One hundred and forty cells are located on a 12 × 12 grid. Values in each block are normalized to the range [−1 1] when plotting this figure.

After learning, synaptic weights between LGN and V1 display spatial structures similar to those observed in recordings of neurons in V1, such as oriented Gabor-like filters and non-oriented blobs. Since both excitatory and inhibitory connections from ON and OFF LGN cells contribute to the responses of simple cells, we use the *synaptic field* (***S***_**f**_) defined as

(18) $$\begin{array}{l} S_{\text{f}}=(A_{\text{ON}}^{\text{u},\text{+}}+A_{\text{ON}}^{\text{u},-})-(A_{\text{OFF}}^{\text{u},\text{+}}+A_{\text{OFF}}^{\text{u},-}) \end{array}$$

to visualize the overall synaptic weights from ON and OFF LGN cells. The synaptic fields of 140 model simple cells that meet the two quality control measures (see the Materials and Methods section) are shown in Figure 5, where each block represents the overall effect of the feedforward connections from ON and OFF LGN cells to a simple cell. Note that although Figure 5 displays spatial patterns that are similar to experimental RFs, strictly they represent the synaptic weights from LGN cells to simple cells, which ignores the early visual processing before LGN. However, the RFs of the model are systematically investigated in the following sections.

In the remaining results, we show that the synaptic weights exhibit several properties that have been observed experimentally, including segregation of ON and OFF sub-regions, push-pull effect, phase-reversed feedback, diverse shapes of simple cell RFs, and contrast invariance of orientation tuning.

### 3.1. Segregated ON and OFF Sub-regions

Hubel and Wiesel found that simple cells in cat striate cortex have spatially separated ON and OFF sub-regions (Hubel and Wiesel, 1962), which was also confirmed by other experimental studies (Jones and Palmer, 1987b; Hirsch et al., 1998; Martinez et al., 2005). However, it is impossible for a model that combines ON and OFF LGN input into a single linear input to explain this important phenomenon. Our model separates ON and OFF LGN input and shows that the learned feedforward excitatory connections from ON and OFF LGN cells to simple cells can account for the segregation of ON and OFF sub-regions of simple cells.

**Figure 6:**
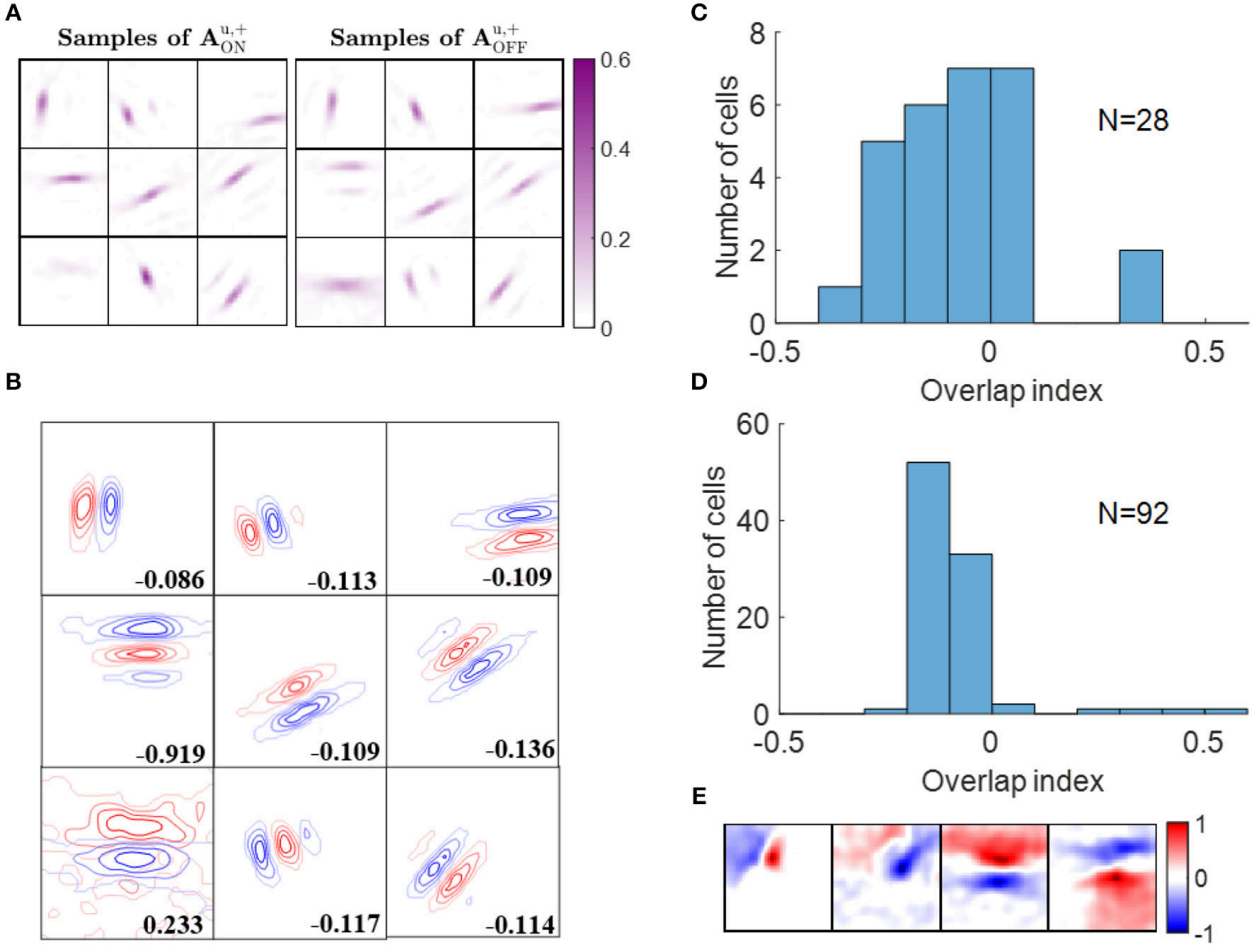
Segregation of ON and OFF sub-regions. **(A)** Some examples of ${\text{A}}_{\text{ON}}^{\text{u},+}$ and ${\text{A}}_{\text{OFF}}^{\text{u},+}$. Each block is a 16 × 16 image that represents 256 excitatory connections from ON or OFF LGN cells to a simple cell. The color magenta represents excitatory connections. **(B)** Red and blue contours represent excitatory connections from ON and OFF LGN cells, respectively. Connections that are smaller than 20% of the maximal connection were removed to only show the substantial weights. The number in each block indicates the overlap index. **(C)** Histogram of the overlap index for simple cells in cat V1. Re-plotted from Figure 3C in Martinez et al. (2005). **(D)** Histogram of the overlap index for model simple cells. **(E)** Synaptic fields of the four simple cells with overlap index larger than 0.1.

ON and OFF excitatory regions of some example simple cells are displayed in Figure 6A. In our model, there are 256 ON LGN and 256 OFF LGN cells located evenly on a 16 × 16 image, so each block in Figure 6A represents 256 excitatory weights from ON or OFF LGN cells to a simple cell. Figure 6A shows that these excitatory connections form spatial patterns such as bars and blobs. Furthermore, a careful examination of the patterns shows that excitatory connections from ON LGN cells are normally adjacent to patterns of excitatory connections from OFF LGN cells, but the two patterns do not overlap, as can be seen when contour plots for the ON and OFF excitatory regions are overlaid in Figure 6B.

We quantified the segregation of ON and OFF sub-regions using the overlap index (defined in the Materials and Methods section). The histogram of the overlap index for simple cells in an experimental study (Martinez et al., 2005) is re-plotted in Figure 6C. Consistent with the experimental data, 88 out of 92 model simple cells had an overlap index smaller than 0.1 (Figure 6D), which indicates that the ON and OFF sub-regions are well-separated in a large majority of the population. The synaptic fields of simple cells whose overlap indices are larger than 0.1 are shown in Figure 6E, revealing that most of them have low spatial frequencies.

### 3.2. Push-Pull Effect

Simple cells are also found to have push-pull responses; i.e., if one contrast polarity excites a cell, the opposite contrast polarity tends to inhibit it (Jones and Palmer, 1987b; Ferster, 1988; Hirsch et al., 1998; Martinez et al., 2005). Even though this effect has been observed in many experimental studies, to our knowledge there has not been a learning model proposed that can explain how this effect emerges. Again, a model that separates ON and OFF LGN input is necessary to investigate the emergence of the push-pull effect. In this section, we show that the push-pull effect for simple cells naturally emerges as a result of neural learning.

**Figure 7:**
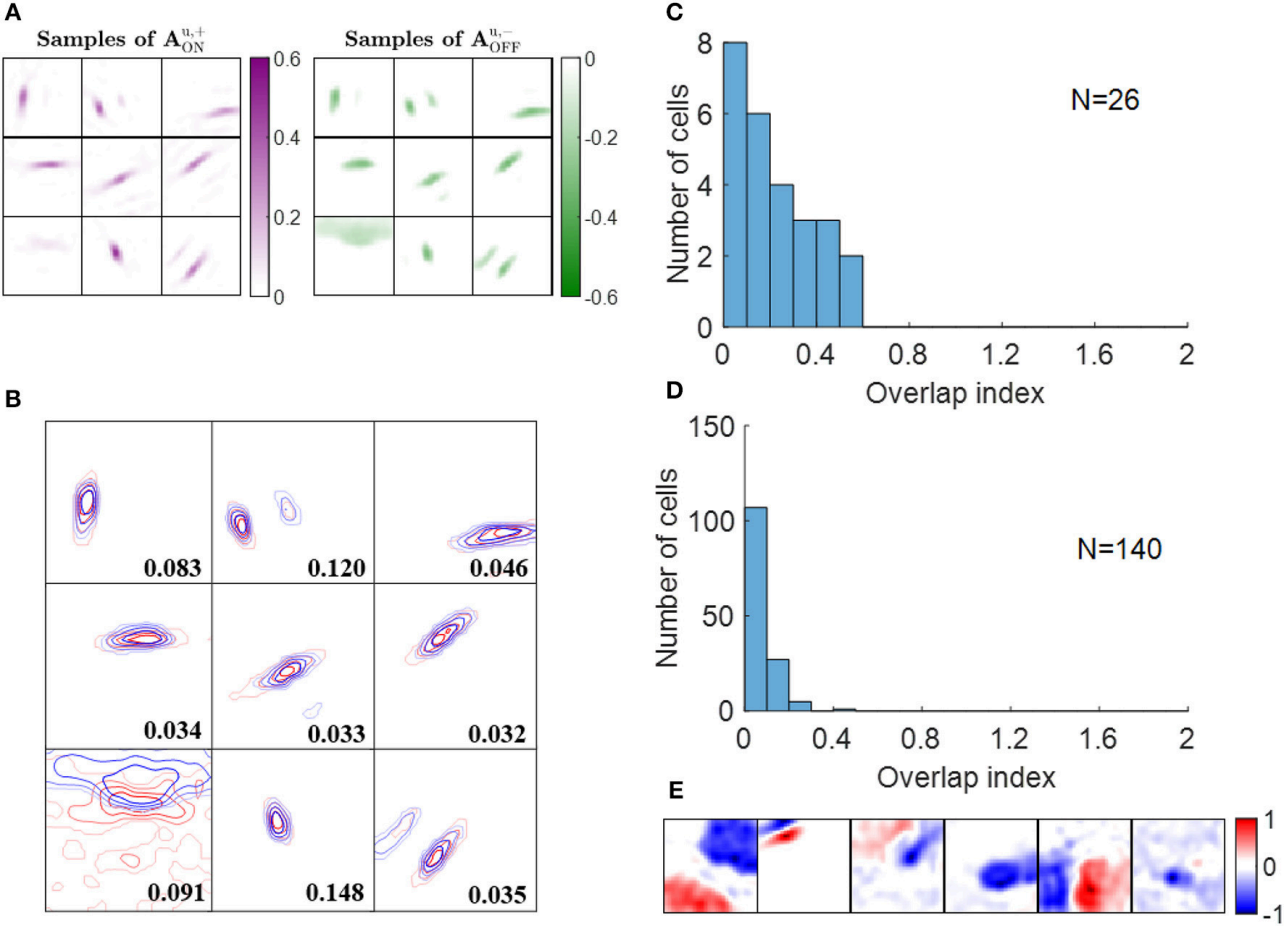
Push-pull effect. **(A)** Some examples of ${\text{A}}_{\text{ON}}^{\text{u},+}$ and ${\text{A}}_{\text{OFF}}^{\text{u},-}$. Each block on the left is a 16 × 16 image that represents 256 excitatory connections from ON LGN cells to a simple cell. Each block on the right represents inhibitory connections from OFF LGN cells to a simple cell. The color magenta represents excitatory connections; the color green represents inhibitory connections. **(B)** Red and blue contours represent excitatory connections from ON LGN cells (${\text{A}}_{\text{ON}}^{\text{u},+}$) and inhibitory connections from OFF LGN cells (${\text{A}}_{\text{OFF}}^{\text{u},-}$), respectively. Connections that are smaller than 20% of the maximal connection were removed to only show substantial weights. The number in each block indicates the push-pull index. **(C)** Histogram of the push-pull index for simple cells in cat V1. Re-plotted from Figure 4B in Martinez et al. (2005). **(D)** Histogram of the push-pull index for model simple cells. **(E)** Synaptic fields of the six simple cells with push-pull index larger than 0.2.

Some examples of ON excitatory and OFF inhibitory synaptic weights (${\text{A}}_{\text{ON}}^{\text{u},+}$ and ${\text{A}}_{\text{OFF}}^{\text{u},-}$, respectively) are shown in Figure 7A. The patterns of ${\text{A}}_{\text{ON}}^{\text{u},+}$ are similar to the ones of ${\text{A}}_{\text{OFF}}^{\text{u},-}$ and they are located at similar locations, as can be seen from the highly overlapped contours in Figure 7B. However, the degree of overlap is different between the examples.

Analogous results to the above also hold for learned excitatory connections from OFF LGN cells, ${\text{A}}_{\text{OFF}}^{\text{u},+}$, and inhibitory connections from ON LGN cells, ${\text{A}}_{\text{ON}}^{\text{u},-}$ (data not shown).

We then quantified the push-pull effect using push-pull index (defined in the Materials and Methods section). Both the histograms of push-pull index for experimental data (Figure 7C) and model simple cells (Figure 7D) peaked near zero and showed an decreasing trend. Model simple cells showed even stronger push-pull index with more simple cells having push-pull index close to zero. The synaptic fields of simple cells with push-pull indices larger than 0.2 are shown in Figure 7E, showing that most of them have low spatial frequencies.

### 3.3. Phase-Reversed Feedback

The experimental study of Wang and colleagues suggests that the synaptic feedback from V1 to LGN is phase-reversed with respect to the feedforward connections (Wang et al., 2006). For example, the connection from a simple cell to an ON-center LGN cell will be excitatory if the ON-center is aligned in visual space to the OFF sub-field of simple cell (i.e., phase-reversed). Conversely, if the ON-center is aligned to the ON sub-field of the simple cell, the connection will be inhibitory. Our learning model with separate ON and OFF LGN cells enables us to investigate the feedback effect from simple cells to LGN cells. In this section, we show that phase-reversed feedback arises in the structures of learned connections.

Feedback from simple cells to LGN cells occurs via both excitatory connections, ${\text{A}}_{\text{x}}^{\text{d},+}$, and inhibitory connections, ${\text{A}}_{\text{x}}^{\text{d},-}$, with the overall effect characterized by ${\text{A}}_{\text{x}}^{\text{d}}={\text{A}}_{\text{x}}^{\text{d},+}+{\text{A}}_{\text{x}}^{\text{d},-}$, where x = ON or OFF depending on the type of LGN cell. Therefore, the overall feedback to ON LGN cells, denoted as ${\text{A}}_{\text{ON}}^{\text{d}}$, can be represented by ${\text{A}}_{\text{ON}}^{\text{d}}={\text{A}}_{\text{ON}}^{\text{d},+}+{\text{A}}_{\text{ON}}^{\text{d},-}$. Similarly, ${\text{A}}_{\text{OFF}}^{\text{d}}={\text{A}}_{\text{OFF}}^{\text{d},+}+{\text{A}}_{\text{OFF}}^{\text{d},-}$ represents the overall feedback to OFF LGN cells.

**Figure 8:**
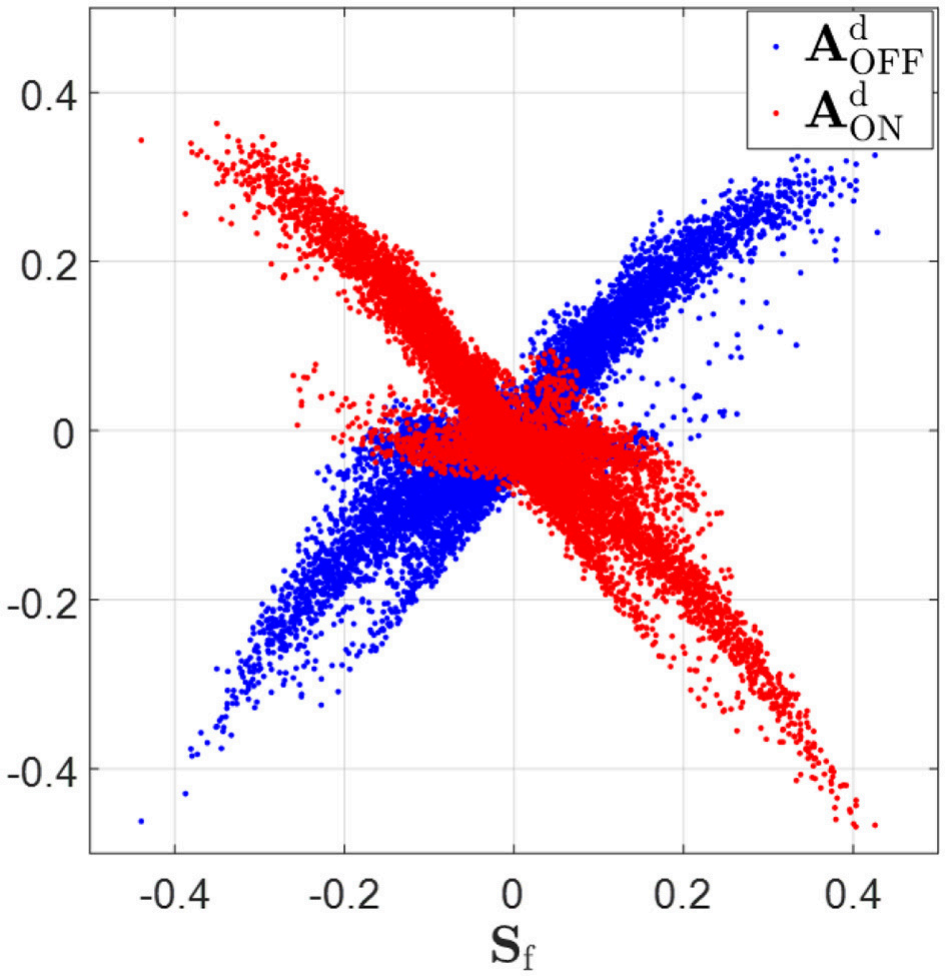
Synaptic fields, **S**_f_ (defined in Equation 18), vs. feedback to ON and OFF LGN cells, ${\text{A}}_{\text{ON}}^{\text{d}}$ and ${\text{A}}_{\text{OFF}}^{\text{d}}$. **S**_f_ is highly positively correlated with ${\text{A}}_{\text{OFF}}^{\text{d}}$ (correlation coefficient *r* = 0.90) and **S**_f_ is highly anti-correlated with ${\text{A}}_{\text{ON}}^{\text{d}}$ (correlation coefficient *r* = −0.92). When **S**_f_ is greater than zero, ${\text{A}}_{\text{OFF}}^{\text{d}}$ tends to be greater than zero and ${\text{A}}_{\text{ON}}^{\text{d}}$ tend to be smaller than zero. On the contrary, ${\text{A}}_{\text{OFF}}^{\text{d}}$ tends to be smaller than zero and ${\text{A}}_{\text{ON}}^{\text{d}}$ tends to be greater than zero if **S**_f_ is negative.

The ON and OFF sub-fields of simple cells receptive fields are characterized by the positive and negative regions of the synaptic field defined in Equation (18). The scatter plots in Figure 8 show that relationship expected from phase-reversed feedback. **S**_f_ is highly positively correlated with ${\text{A}}_{\text{OFF}}^{\text{d}}$ (correlation coefficient *r* = 0.90), while **S**_f_ is highly anti-correlated with ${\text{A}}_{\text{ON}}^{\text{d}}$ (correlation coefficient *r* = −0.92). According to the figure, wherever **S**_f_ is positive, indicating the ON sub-field, the feedback to ON LGN cells, ${\text{A}}_{\text{ON}}^{\text{d}}$, is very likely to be negative and the feedback to OFF LGN cells, ${\text{A}}_{\text{OFF}}^{\text{d}}$, tends to be positive; however, wherever **S**_f_ is negative, indicating the OFF-field, the converse is true: the feedback to ON LGN cells, ${\text{A}}_{\text{ON}}^{\text{d}}$, is very likely to be positive and the feedback to OFF LGN cells, ${\text{A}}_{\text{OFF}}^{\text{d}}$, tends to be negative. This corresponds to a phase-reversed feedback from V1 to LGN.

**Figure 9:**
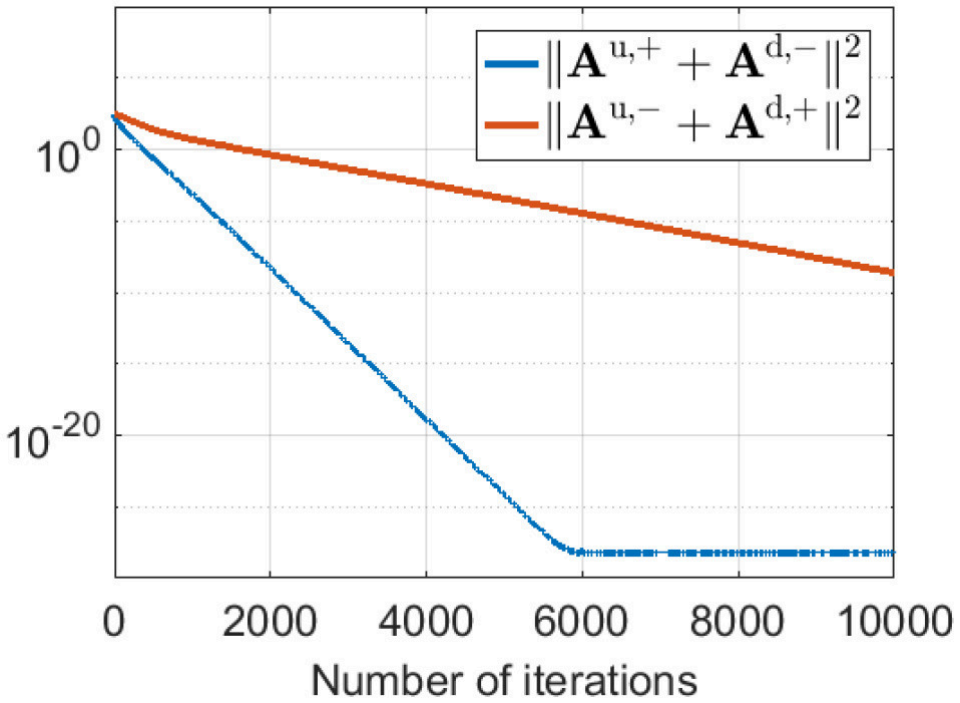
||**A**^u,+^ + **A**^d,−^||^2^ and ||**A**^u,−^ + **A**^d,+^||^2^ during pre-development when white noise is used as the input. The difference between **A**^u,+^ and −**A**^d,−^ (blue line) decreases to zero very quickly during learning. Similarly, the difference between **A**^u,−^ and −**A**^d,+^ (red line) reduces to zero quickly, although somewhat slower than the blue line.

This phase-reversed feedback from V1 to LGN can be explained by the learning dynamics of LGN and simple cells described in Equation 8. The learning rule shows that **A**^u,+^ and **A**^d,−^ are updated with the same magnitude of synaptic change but opposite in sign (and are normalized with the same norm *l*_1_). Similarly, **A**^u,−^ and **A**^d,+^ are updated with the same magnitude of synaptic change but opposite in sign (and are normalized with the same norm *l*_2_). These anti-symmetries are a consequence of having Hebbian learning for the forward weights and anti-Hebbian learning for the feedback weights. In both cases the magnitude of weight change is proportion to the production of pre- and post-synaptic spike rates, but the sign of the change is opposite. The anti-symmetry arises because roles of pre- and post-synaptic rates are interchanged in forward vs. feedback directions, in combination with the sign change. Simulation results show that **A**^u,+^ converges to −**A**^d,−^ and **A**^u,−^ converges to −**A**^d,+^ even during pre-development when white noise is used as the input to the model, as illustrated in Figure 9.

### 3.4. The Diversity of Model Receptive Fields Resembles That Observed Experimentally for Simple Cells

In this section, we show that the range of spatial structures of RFs of our model have a close resemblance to experimental data.

RFs were calculated from the model by simulating experiments in which Gaussian white noise is presented as a visual stimulus, and the spike triggered average is used to estimate RFs. As the presentation of white noise may cause adaptive effects in the early stages visual system relative to natural images, we considered two versions of the model, one with the standard pre-whitening filter (Equation 9) modeling center-surround processing, and a second without pre-whitening in which the filter is replaced by a low-pass filter (Equation 11) with the same upper cut-off frequency as pre-whitening filter. We use *pre-whitened RFs* and *low-pass RFs* to represent of simple cell RFs estimated using the pre-whitening filter and low-pass filter.

**Figure 10:**
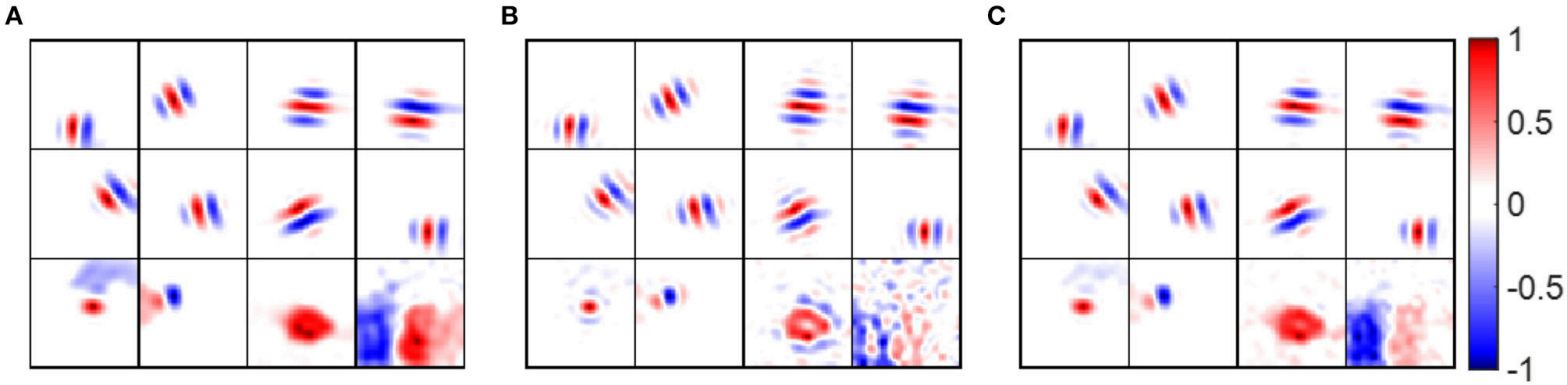
Receptive fields of example model cells. Values of each block are normalized to the range [−1 1] when plotting the figure. **(A)** Synaptic fields of example model cells. **(B)** Pre-whitened RFs of example model cells. The pre-whitening filter described in Equation (9) was used to filter white noise stimuli. **(C)** Low-pass RFs of example model cells. The low-pass filter described in Equation (11) was used to filter white noise stimuli.

Some examples of pre-whitened RFs, low-pass RFs and synaptic fields are shown in Figure 10, which shows that pre-whitened RFs and low-pass RFs are similar to synaptic fields. However, pre-whitened RFs tend to have more and thinner stripes, which indicates a narrower tuning to a somewhat higher spatial frequency. For a simple cell tuned to very low spatial frequencies (bottom right blocks), the RF recovered with pre-whitening was a poor match to the original synaptic field, but for RF recovered with low-pass filtering it was fair.

**Figure 11:**
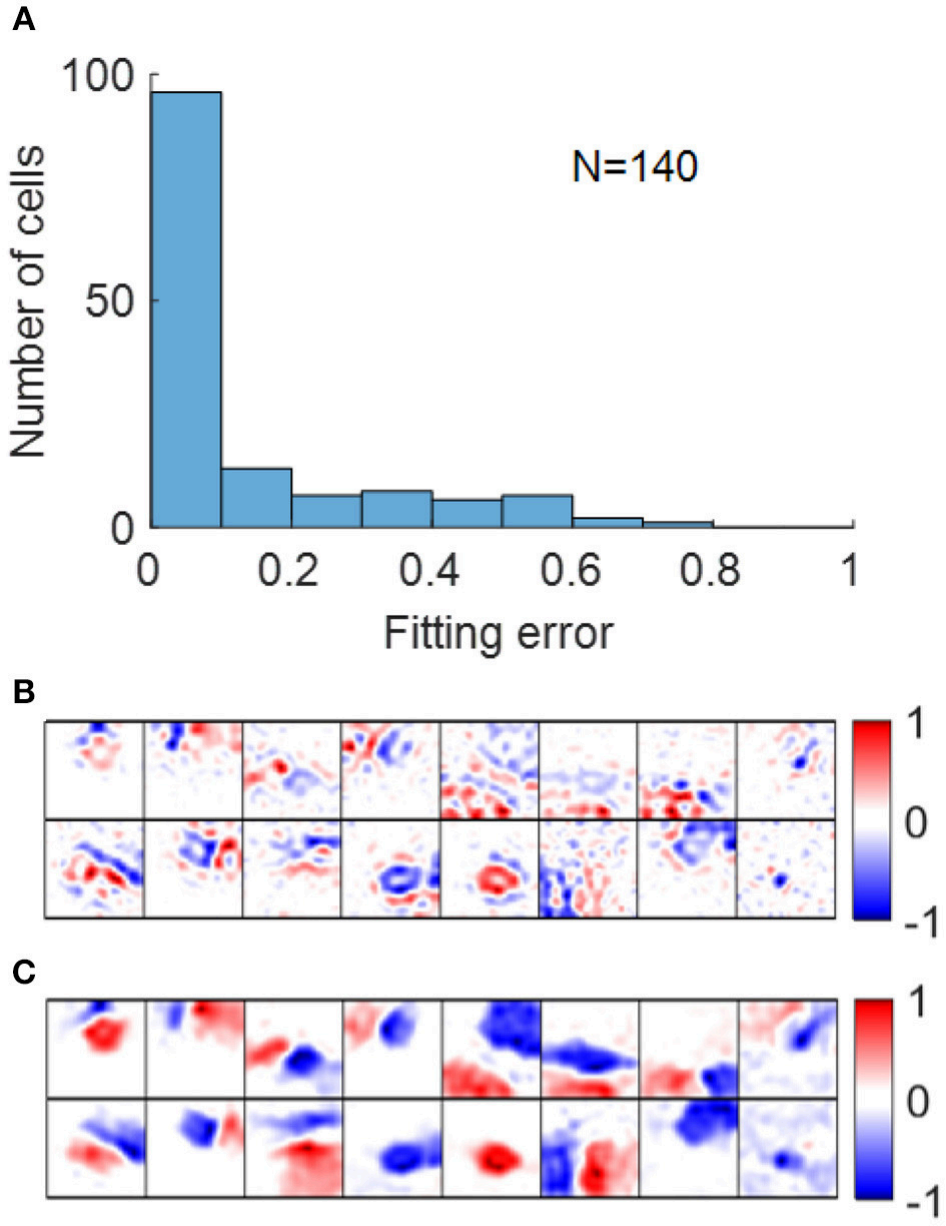
**(A)** Histogram of Gabor fitting errors for pre-whitened RFs. **(B)** Pre-whitened RFs that has fitting error larger than 40%. **(C)** Synaptic fields of the corresponding cells in **(B)**.

Early studies show that RFs of simple cells can be well-described by 2D Gabor functions described in Equation (12) (Jones and Palmer, 1987a; Ringach, 2002). For our model, most RFs could be well-fitted by Gabor functions with suitable choices of parameters with small fitting errors, as shown in Figure 11A. Note that although the fitting error of blob-like RFs might be low, the parameter choices are not necessarily reasonable, in that they are poorly constrained and the process of Gabor fitting imposes an a priori hypothesis that the RF is a 2D-Gabor function even though it is clearly not Gabor-like. The pre-whitened RFs with fitting errors larger than 40% (Figure 11B) are cells whose synaptic fields have low spatial frequencies (Figure 11C), because pre-whitened RFs of these cells matched poorly to the original synaptic fields (Figure 10B). Low-pass RFs of all 140 selected model cells have fitting errors smaller than 40% with 132 of them having fitting errors smaller than 20% (data not shown).

Using fitted parameters of Gabor functions, Ringach constructed a scatter plot of *n*_*x*_ = σ_*x*_*f*_*s*_ vs. *n*_*y*_ = σ_*y*_*f*_*s*_ to analyze the spatial structures of RFs in V1 over the population (Ringach, 2002). Such plots have subsequently been used by many researchers to investigate the distributions of model simple cell RFs (Rehn and Sommer, 2007; Wiltschut and Hamker, 2009; Zylberberg et al., 2011). *n*_*x*_ and *n*_*y*_ are the width and length of the Gabor function measured in the number of cycles of the spatial frequency (i.e., across and along the stripes). Ringach noted that blob-like RFs are mapped to points near the origin, while RFs with elongated sub-regions are mapped to points away from the origin (Ringach, 2002). In addition, *n*_*x*_ and *n*_*y*_ are directly related with the half-magnitude spatial frequency bandwidth Δ*f* and orientation bandwidth Δθ of the fitted Gabor function,

(19) $$\begin{array}{l} \Delta f:=h(n_{x})=\log_{2}\left(\frac{1+\frac{\sqrt{2\ln2}}{2\pi n_{x}}}{1-\frac{\sqrt{2\ln2}}{2\pi n_{x}}}\right)\text{ in octaves}\\ \Delta \theta :=g(n_{y})=2\arctan\left(\frac{\sqrt{2\ln2}}{2\pi n_{y}}\right)\text{ in degrees}. \end{array}$$

Both *h*(*n*_*x*_) and *g*(*n*_*y*_) are monotonically decreasing functions; i.e., the larger *n*_*x*_ and *n*_*y*_, the smaller Δ*f* and Δθ. Note that *h*(*n*_*x*_) is not well-defined when $n_{x}<\sqrt{2\ln\;2}/2\pi \;\left(\approx 0.13\right)$, i.e., when the lower half-magnitude frequency do not exist. This corresponds to the region in which Gabor fitting gives ambiguous fits for parameters like spatial frequency and orientation, because oriented RFs with low spatial frequency might lie in this region as well.

**Figure 12:**
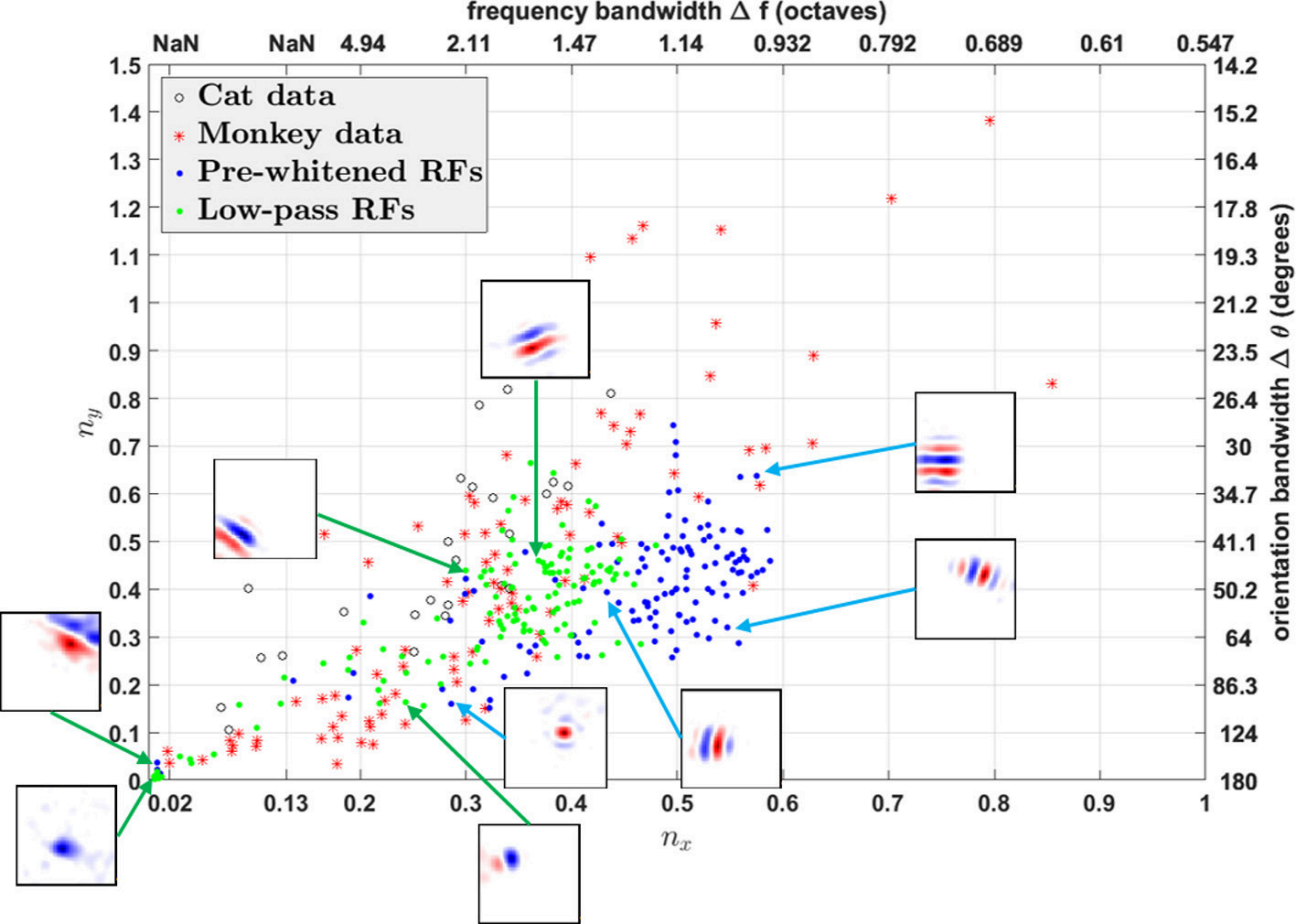
*n*_*x*_ vs. *n*_*y*_. Comparison of RFs of the model with experimentally recorded data for cat simple cells and monkey simple cells. Open circles: 25 cat simple cells from Table 1 in Jones and Palmer (1987a) re-plotted in the (*n*_*x*_, *n*_*y*_) plane; red stars: 93 monkey simple cells in Ringach (2002); blue dots: pre-whitened RFs using the pre-whitening filter described in Equation (9); green dots: low-pass RFs using the low-pass filter described in Equation (11). The axes on the top and right represent frequency and orientation bandwidths of fitted Gabor functions computed using Equation (19). Some examples of RFs are displayed in the inset sub-plots. Data points of estimated RFs with fitting errors > 40% were excluded, which gave 124 data points for pre-whitened RFs and 140 data points for low-pass RFs.

We plot *n*_*x*_ vs. *n*_*y*_ and Δ*f* vs. Δθ for RFs obtained from both the model and experimental studies in Figure 12. However, the different pre-processing filters for white noise stimuli have a dramatic influence on the distributions of *n*_*x*_ vs. *n*_*y*_, shifting the distribution for low-pass RFs to the left of pre-whitened RFs, in closer agreement to the experimental data. As mentioned earlier, pre-whitened RFs tend to have more stripes relative to the low-pass RFs, so they are mapped to points away from the origin compared to low-pass RFs. In addition, the distribution of low-pass RFs is continuous from the origin, while there is a gap between points near the origin and points away from the origin for pre-whitened RFs. The inset sub-plots of Figure 12 show that data points near the origin might be orientated RFs with low spatial frequencies and blob-like RFs might not be necessarily mapped to points near the origin.

In general, oriented RFs are well-described by Gabor functions and low-pass RFs better resemble the distribution of experimental data compared with pre-whitened RFs.

### 3.5. Contrast Invariance of Orientation Tuning

**Figure 13:**
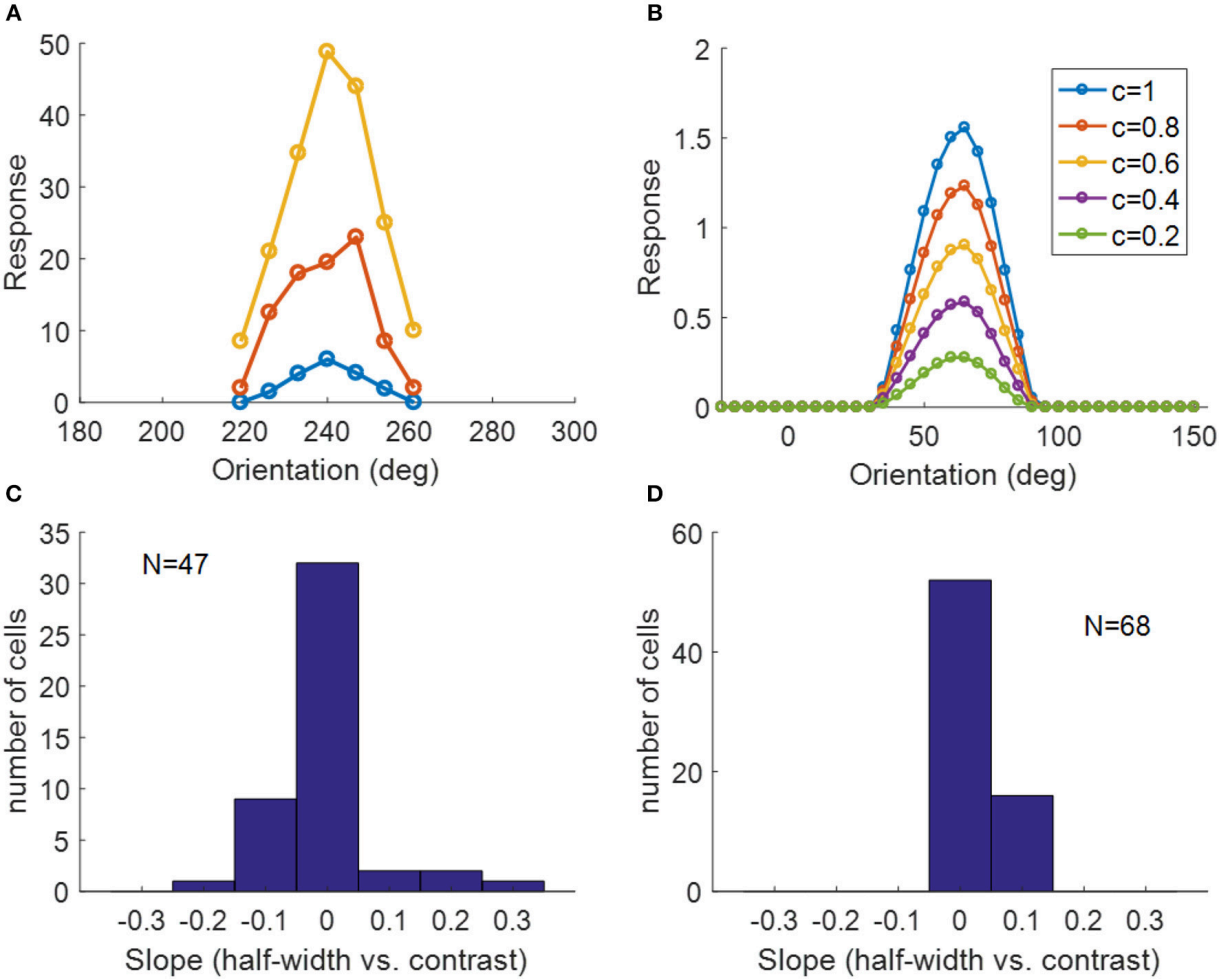
Contrast invariance of orientation tuning. **(A)** Contrast invariant orientation tuning curves of a simple cell in cat V1. Re-plotted from Figure 3A in Skottun et al. (1987). Different colors represent different contrasts. **(B)** Contrast invariant orientation tuning curves of a cell in our model. *c* = 1 and *c* = 0.2 correspond to the high and low contrast, respectively. **(C)** Histogram of the slope of half-height bandwidth vs. contrast for V1 population in ferret. Re-plotted from Figure 3B in Alitto and Usrey (2004). **(D)** Histogram of the slope of half-height bandwidth vs. contrast for model simple cells.

Another important property of simple cells is contrast invariance of orientation tuning; i.e., the width of the orientation tuning curve is maintained when the contrast of the stimulus changes, as demonstrated in Figure 13A. The orientation tuning curves with various stimulus contrasts for a model simple cell are shown in Figure 13B, where the bandwidths of each curve remain the same while the responses become larger when the stimulus contrast increases. For a study of contrast invariance of V1 population in ferret, the histogram of the slope of the linear fit of half-width bandwidth vs. contrast (Figure 13C) showed that most cells were contrast invariant with the slope close to zero (Alitto and Usrey, 2004). Figure 13D shows that most model cells have the slope around zero, which is consistent with experimental data.

## 4. Discussion

### 4.1. Relationship With Sparse Coding

Sparse coding has been successful in modeling simple cell receptive fields (RFs) and has been used by many researchers over the past years. Our model is based on an algorithm that efficiently implements sparse coding (Rozell et al., 2008), and is therefore closely related to the original concept of sparse coding (Olshausen and Field, 1996).

If we define **A** as a 2*N* × *M* matrix that represents the overall effect caused by excitatory and inhibitory connections from 2*N* LGN cells to *M* simple cells, we have **A** = **A**^u,+^ + **A**^u,−^. The dynamics of simple cells described in Equation (7) can be rewritten as

(20) $$\begin{array}{l} \tau_{\text{C}}{\dot{v}}^{\text{C}}=-v^{\text{C}}+A^{T}(s^{\text{L}}-s_{\text{b}})+s^{\text{C}}. \end{array}$$

As illustrated in Figure 9, **A**^u,+^ → −**A**^d,−^ and **A**^u,−^ → −**A**^d,+^ during learning. Therefore, we have **A**^d,−^ + **A**^d,+^ = −**A**^u,+^ − **A**^u,−^ = −**A**. The dynamics of LGN cells described in Equation (5) can be rewritten as

(21) $$\begin{array}{l} \tau_{\text{L}}{\dot{v}}^{\text{L}}=-v^{\text{L}}+x-As^{\text{C}}+s_{\text{b}}. \end{array}$$

If the columns of **A** are seen as the basis vectors of a generative model, **As**^C^ can be seen as the linear reconstruction of the input using learned basis vectors and thus **x** − **As**^C^ represents the residual error, which is similar to **r** of the sparse coding formulation given in Equation (2). Therefore, the residual error used to update the basis vectors of the original sparse coding model is represented by the responses of LGN cells in our model.

To incorporate Dale's law, non-negative connections, **A**^u,+^, and non-positive connections, **A**^u,−^, are employed in our model to represent the positive and negative elements of **A**. **A**^u,+^ and **A**^u,−^ are not co-active in general, which suggests that ${\text{A}}^{\text{u},+}\approx {\left[\text{A}\right]}_{+}$ and ${\text{A}}^{\text{u},-}\approx {\left[\text{A}\right]}_{-}$, where [ · ]_+_ preserves the positive elements and sets negative elements to zero and [ · ]_−_ preserves the negative elements and sets positive elements to zero.

In other words, our model is essentially a variant of sparse coding that employs separate connections to learn the positive and negative part of the overall connections.

### 4.2. Relationship With Predictive Coding

Our model is a hierarchical model with feedforward and feedback connections based on a locally competitive algorithm (Rozell et al., 2008). The structure of our model is essentially very similar to that of predictive coding models. To be more specific, the feedback from the second-layer neurons reconstruct the input. The residual error is computed at the first layer and then propagated to the second layer via feedforward connections.

Although our model presented here and the predictive coding model of Jehee and Ballard (2009) can explain phase-reversed feedback, the models differ in several respects. First, sparse coding in our model is simply realized by the threshold of the rectifying function of firing rates for simple cells and this simple mechanism leads to simple neural circuits. Second, compared to the mechanism for determining simple cell responses one by one in their model, our model computes the responses in parallel. Third, our model generates diverse types of RFs that correspond well to experimental data. Finally, the phase-reversed effect is simply accounted for by the special pattern of learned connections, which also explains the segregation of ON/OFF sub-regions and push-pull effect for simple cells.

### 4.3. The Function of Spontaneous Activity

In the model proposed here, the dynamics of LGN cells described in Equation (5) has the background firing rate, *s*_b_, as part of the input to LGN cells. This spontaneous firing rate introduces a shift of the operating point for LGN cells. Given the responses of simple cells, **s**^C^, **x** − **As**^C^ in Equation (21) represents the reconstruction residual error between the input and reconstruction. The residual error gives the difference between the real input and the representation produced by the model and it can be either positive or negative. To code for the signed quantities (residual error), Ballard and Jehee carried out a case-by-case study, leading to very complicated neural circuits (Ballard and Jehee, 2012). However, our model has a straightforward method for the implementation of solving signed quantities. The background firing rate, *s*_b_, in Equation (5) increases the residual errors by *s*_b_. Therefore, the membrane potential of LGN cell, **v**^L^, represents the residual error shifted up by *s*_b_. The threshold function in Equation (5) gives the firing rate of the LGN cell and it preserves the residual error in the interval of [−*s*_b_, ∞], which preserves the information of whether the model under-estimates or over-estimates the input stimuli and forces the connections to evolve through learning in the correct direction. In Equation (7), which describes simple cell dynamics, the effect of the spontaneous firing rate, *s*_b_, is removed by ${\text{v}}_{\text{leak}}^{\text{C}}$, a homeostatic mechanism employed by simple cells to maintain resting membrane potentials when there is no external input. The local learning rule described by Equation (8) also eliminates the effect of the spontaneous firing rate by subtracting it. The use of spontaneous firing rate makes the model much simpler and offers a new approach for solving the problem of signed quantities (residual errors). Experimental evidence shows that thalamocortical neurons can fire with bursts of action potentials without any synaptic input (Kandel et al., 2013), which suggests that the spontaneous firing activities might be used to encode the difference between input and feedback information.

### 4.4. Pre-processing of the Early Visual System

Atick and Redlich suggest that the retinal goal is to whiten the visual input up to a transition frequency such that input noise can also be suppressed (Atick and Redlich, 1992). The pre-whitening filter (Equation 9) approximately whitens the natural scenes up to the cut-off frequency.

However, for pre-processing white noise stimuli, two hypotheses are considered here. First, the filtering process of the early visual system can be described by the pre-whitening filter (Equation 9) whether or not the visual stimuli are natural scenes. Second, the early visual system is adaptive such that the visual stimuli are whitened up to a cut-off frequency. In this case, a low-pass filter (Equation 9) should be used, because white noise stimuli are already whitened across all frequencies. Our results suggest that estimated RFs using low-passed white noise match the experimental data much better than estimated RFs using pre-whitened white noise. Further investigation of how visual stimuli are processed before they are fed to the visual cortex is needed to better understand the properties of simple cells.

### 4.5. The Role of *l*_1_ and *l*_2_

Each column of **A**^u,+^ and **A**^d,−^ is normalized to norm *l*_1_ and each column of **A**^u,−^ and **A**^d,+^ is normalized to norm *l*_2_. In other words, *l*_1_ represents the overall strength of feedforward excitatory connections and feedback inhibitory connections while *l*_2_ represents the overall strength of feedforward inhibitory connections and feedback excitatory connections. The results shown in this paper are based on *l*_1_ = 1 and *l*_2_ = 1; i.e., the strength of feedforward excitatory connections is equivalent to feedforward inhibitory connections, which leads to a strong push-pull effect in Figure 7D. If *l*_2_ is smaller than *l*_1_, the push-pull effect will be weaker and the distribution of the push-pull index will shift to the right. In addition, reducing *l*_2_ results in more blob-like receptive fields (data not shown).

### 4.6. Neural Circuits

Biologically realistic neural models can provide deeper insights into how real neural circuits function. The model proposed here contains a number of features that correspond to those in its biological counterpart, namely in terms of ON and OFF channels for LGN cells, positive neuronal responses, local computation, local learning rule, existence of feedback, and obedience to Dale's law.

In addition, our model incorporates inhibitory effects between LGN cells and cortical simple cells. As pointed out in the Materials and Methods section, for simplicity, inhibitory effects are implemented by direct inhibitory connections between two layers. However, in reality, long-range inhibitory effects should be implemented via interneurons that have inhibitory synapses. In this section, we will discuss several neural circuits of implementing inhibitory connections of our model.

**Figure 14:**
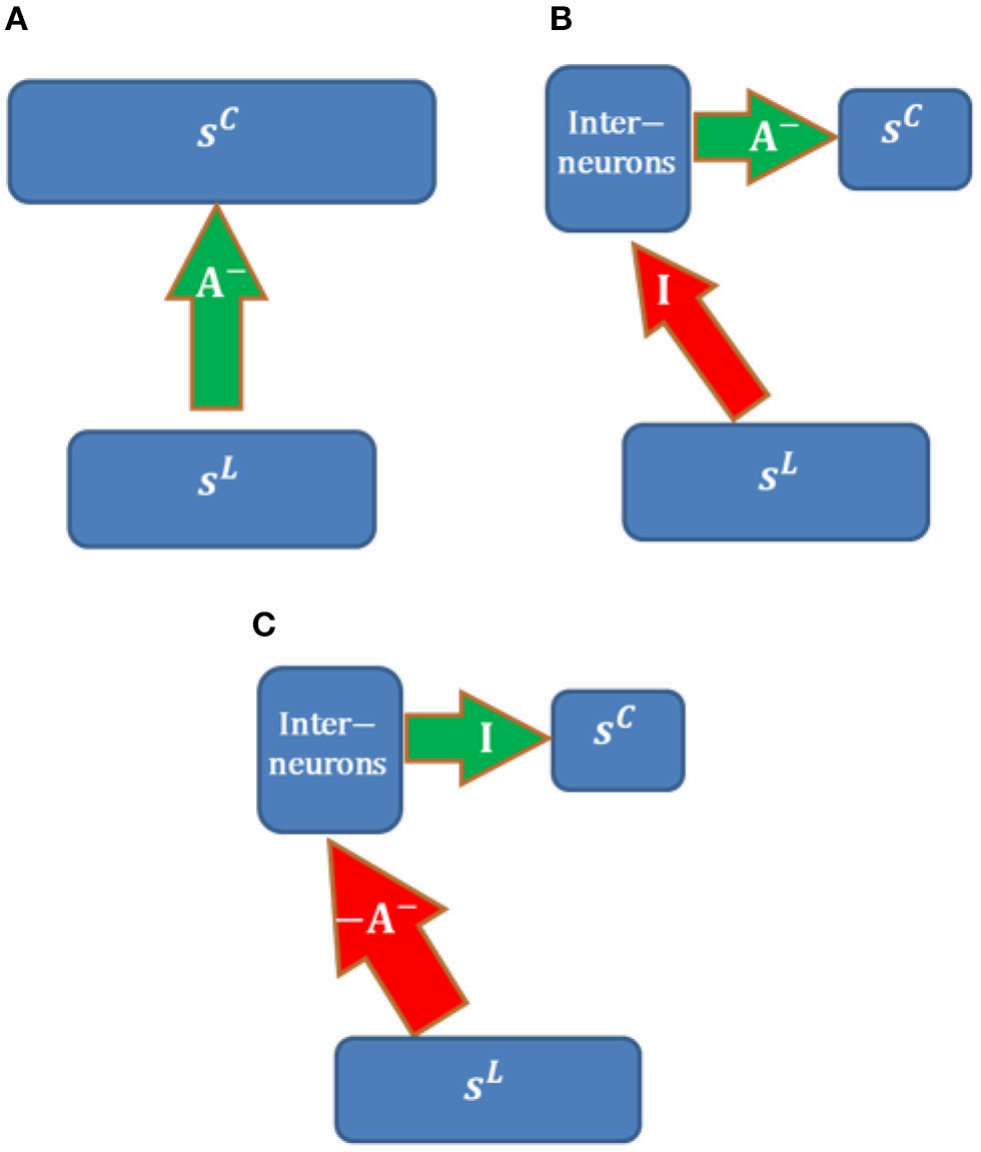
Possible neural circuits for implementing long-range inhibition. Red and green arrows represent excitatory and inhibitory connections. **(A)** Direct long-range inhibition. **(B)** Circuit I. **(C)** Circuit II.

Possible neural circuits that may be used to implement long-range inhibition are displayed in Figure 14. Assume that the overall inhibitory effects from LGN cells (with activity **s**^L^) to cortical simple cells (with activity **s**^C^) can be represented by inhibitory connections, **A**^−^, between populations. We also assume that the learning rule of **A**^−^ is local, i.e., that only depends on the responses of two populations (**s**^L^ and **s**^C^). Long-range inhibition in our model is implemented via direct inhibitory connections, which is not biologically realistic (Figure 14A).

The circuit in Figure 14B implements inhibitory connections, **A**^−^ (with non-positive weights), via a population of interneurons that have inhibitory connections, **A**^−^, with cortical simple cells. LGN cells are connected to interneurons via long-range identical excitatory connections, **I**; i.e., the interneurons copy the responses of LGN cells. For this structure, long-range excitatory connections, **I**, are fixed while **A**^−^ are learned using the same learning rule in Figure 14A. In this case, the learning rule of **A**^−^ is still local because the responses of interneurons are just **s**^L^ and the model is still biologically plausible in terms of the local learning rule. Furthermore, the RFs of interneurons in the same layer as cortical simple cells should be LGN-like. Though V1 cortical cells with blob-like RFs were found in different species (Kretz et al., 1986; Jones and Palmer, 1987a; Hawken et al., 1988; Muly and Fitzpatrick, 1992; Chapman and Stryker, 1993; Ringach, 2002), we are not sure whether this neural circuit is the most likely candidate because the fixed identical connection between LGN cells and the interneurons seems artificial unless they can be learned.

Figure 14C shows another possible neural circuit for implementing **A**^−^. LGN Cells are connected to interneurons via long-range excitatory connections, −**A**^−^. There is a one-to-one mapping between interneurons and cortical simple cells. In this case, the overall effect from LGN cells to simple cells is equivalent to **A**^−^. In addition, the RFs of inhibitory interneurons should resemble simple cells and show orientation tuning since the learned **A**^−^ has spatial structures such as oriented bars, which is consistent with the smooth simple cells found in cat V1 of the experimental study (Hirsch et al., 2003). The positive connections −**A**^−^ can be learned by Hebbian learning and the identical connections between interneurons and cortical simple cells can be learned by anti-Hebbian learning. Therefore, this neural circuit is more feasible than than the circuit in Figure 14B.

### 4.7. Discrepancies Between Model and Experimental Data

Our model can capture the most significant features of experimental phenomena such as the segregation of ON and OFF sub-regions, push-pull effect and contrast invariance of orientation tuning. However, there are also discrepancies between the distributions of model and experimental data. In general, the histograms of experimental data (Figures 6C, 7C, 13C) are wider than model data (Figures 6D, 7D, 13D), which shows that experimental data is more diverse. One possible explanation is that model cells in this paper are only a subset of the rich repository of real cortical cells. Furthermore, choices of free parameters in the model might also lead to different results.

## 5. Conclusion

In this paper, we presented a biologically plausible model of LGN-V1 pathways to account for many experimental phenomena of V1. We found that the segregation of ON/OFF sub-regions of simple cells, push-pull effect, and phase-reversed cortico-thalamic feedback can all be explained by the structure of learning connections when the model incorporates ON and OFF LGN cells and is trained using natural images. Furthermore, the model can produce diverse shapes of receptive fields and contrast invariance of orientation tuning of simple cells, consistent with experimental observations.

## Data Availability

The datasets generated for this study are available on request to the corresponding author.

## Author Contributions

All authors listed have made a substantial, direct and intellectual contribution to the work, and approved it for publication.

### Conflict of Interest Statement

The authors declare that the research was conducted in the absence of any commercial or financial relationships that could be construed as a potential conflict of interest.
